# A Proteogenomic Approach to Discover Novel lncRNA-Derived Microproteins and Their Potential Clinical Utility in Hepatocellular Carcinoma

**DOI:** 10.1016/j.mcpro.2026.101584

**Published:** 2026-05-14

**Authors:** Bingwu Li, Kandarp Joshi, Dan Ohtan Wang

**Affiliations:** 1 Biology Program, Division of Science, New York University Abu Dhabi, Abu Dhabi, UAE; 2 Department of Oncology, Sidney Kimmel Comprehensive Cancer Center, School of Medicine, Johns Hopkins University, Baltimore, Maryland, USA; 3 Johns Hopkins All Children’s Hospital, St Petersburg, Florida, USA

**Keywords:** Biomarker, HCC, lncPep, proteogenomic

## Abstract

Microproteins (i.e., peptides) are increasingly recognized for their functions in versatile biological contexts, but their clinical relevance and utility remain largely unexplored. Proteogenomic approaches can accelerate microprotein discovery in clinical samples by integrating proteomic data with genomics and transcriptomics evidence. However, long noncoding RNA (lncRNA)-derived microproteins (lncPeps) remain largely unidentified, resulting in unmatchable MS/MS spectra. To solve this problem, we have used high-quality Ribo-seq translatomic datasets to generate an extensive database of human liver lncRNA-derived open reading frames (lncORFs), which we subsequently applied to proteomics data of tumor–adjacent normal tissue pairs from hepatocellular carcinoma (HCC) patients. Using the new database, we discovered 104 novel lncPeps, including 46 lncPeps differentially expressed between tumor and nontumor tissues, and 13 lncPeps with significant correlation with prognosis. Remarkably, combining the expression of lncPeps with canonical proteins in a LASSO regression model improved predictive performance for recurrence, increasing the AUC by 0.005 to 0.085 across three recurrence time points. These findings suggest that the discovery of lncPeps contributes to our understanding of the molecular heterogeneity and progression of HCC and broadens the range of potential biomarker candidates and treatment targets for the disease.

## Highlights

• A Ribo-seq-based database enabled systematic discovery of human liver lncPeps.
• Identification of 104 novel lncPeps in hepatocellular carcinoma patient tissues.
• Tumor-associated and prognostic lncPeps revealed by proteogenomic analysis.
• lncPeps improve recurrence prediction when integrated with canonical proteins.

## Graphical Abstract

**Figure:**
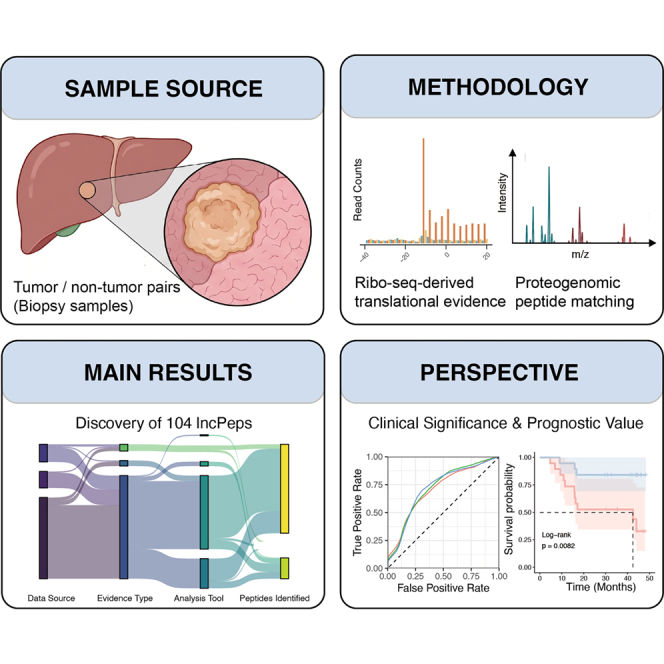

## In Brief

Integrated Ribo-seq and mass spectrometry mapping of the liver cancer “dark proteome” reveals 104 novel microproteins translated from lncRNA open reading frames (lncPeps). This proteogenomic analysis identifies 46 tumor-specific and 13 prognostic lncPeps, whose inclusion in clinical models enhances HCC recurrence prediction by up to 0.085 AUC. These findings establish a robust discovery workflow for noncanonical microproteins, characterizing them as a critical, functional layer of the human proteome with substantial clinical utility for patient management.

Hepatocellular carcinoma (HCC) is the third leading cause of cancer-related mortality worldwide and the fastest-rising cause of cancer-associated deaths in Western countries (1, 2). Although surgical treatment can be effective in the early stages, once HCC develops, the overall 5-year survival rate remains at only 50% to 70% (1). With advances in multi-omics and gene-editing technologies, precision medicine has brought new hopes to patients with HCC. However, the high degree of molecular heterogeneity in HCC presents numerous challenges, including low response rates, drug resistance, and immune evasion (3). To overcome these issues, it is imperative to gain a thorough understanding of the molecular mechanisms underlying tumor heterogeneity. Furthermore, early diagnosis of HCC remains problematic, and the development of clinically useful biomarkers for HCC management has been slow. Alpha-fetoprotein (AFP) has been used as a biomarker in clinical practice for over 6 decades; however, its diagnostic utility remains controversial due to variable sensitivity and specificity (4, 5).

Proteomic analyses have provided critical insights into biomarker discovery and therapeutic strategies. A deeper understanding of the human proteome is essential for deciphering the molecular mechanisms of cancers such as HCC and for advancing precision medicine (6, 7, 8). Despite extensive efforts over the years, a substantial portion of the proteome remains uncharacterized (9). Expanding systematic annotation and functional characterization are crucial for identifying previously unrecognized protein-coding elements, particularly short coding sequences (less than 300 nt) that had been traditionally classified as “noncoding”. A large fraction of these unannotated microproteins may be nested within lncRNAs. In SmProt, a depository of small proteins with experimental evidence of translation, 10.2% of the small ORFs (sORFs) are nested in lncRNA (10). More recent resources further support the widespread translational potential of lncRNAs. LncPepAtlas curated more than 40,000 human translatable lncRNAs by integrating nine direct and indirect lines of evidence, cncRNAdb documented over 1300 translated non-coding RNAs, and OpenProt (v2.0) reported 617,172 alternative proteins, of which 48,620 (7.88%) are derived from ncRNAs (11, 12, 13). These resources highlight the growing translational landscape of lncRNAs and underscore the biological relevance of lncRNA-derived microproteins (lncPeps), which have been implicated in diverse physiological and pathological contexts, including cancer. Several lncPeps have been shown to modulate tumor growth and metastasis by interacting with oncogenic signaling pathways or tumor suppressors (14, 15, 16, 17, 18). In addition, lncPeps have been implicated in immune regulation (19, 20) and contribute to chemotherapy resistance by altering the tumor microenvironment and affecting drug response (20, 21, 22, 23, 24).

Recent reports have also demonstrated the potential of lncPeps as biomarkers and therapeutic targets for HCC, offering unique insights into HCC pathogenesis and molecularly targeted therapy. Guo *et al.* computationally predicted an sORF in lncRNA *ZFAS1* using machine learning, which encodes a lncPep that elevates intracellular reactive oxygen species (ROS) production by inhibiting nicotinamide adenine dinucleotide (NAD) dehydrogenase expression and thus promotes cancer cell migration (25). Micropeptide in mitochondria (MPM) interacts with NAD dehydrogenase NDUFA7, inhibiting Mitochondrial complex I activity and ultimately inhibiting the metastasis of HCC cells (26). lncPep KRASIM was identified using Ribo-seq, which interacts with the oncogenic protein KRAS and suppresses HCC cell growth and proliferation (27). lncPep PINT87aa was identified by Ribosome Nascent-chain Complex RNA sequencing (RNC-seq). PINT87aa directly binds to FOXM1 to block *PHB2* transcription, inducing cell cycle arrest and cellular senescence thus playing a tumor-suppressive role (28). lncPep SMIM30 was identified by RIP-seq assay with an antibody against ribosomal protein S6 (RPS6). SMIM30 functions as an adaptor for the membrane anchoring of SRC/YES1 and contributes to its activation and promotes HCC tumorigenesis (29). lncPep Linc013026-68AA, identified through qRT-PCR analysis of polysome fractions, is suggested to play a role in regulating cell proliferation, although its precise mechanism of action remains unclear (30). Another lncRNA, *AC115619*, encodes a lncPep of 22 amino acids (AC115619–22aa) that has been proposed to disrupt the formation of N6-methyladenosine (m6A) methylation complexes, leading to reduced global m6A levels and HCC progression (31). Additionally, the mitochondrial RNase P inhibitory peptide (MRPIP), derived from an lncRNA *AC027045.3*, has been shown to suppress HCC progression by modulating mitochondrial RNA processing pathways (32).

However, lncPeps in the human genome remain largely unannotated by conventional genome annotation tools owing to their noncanonical and noisy nature in proteomic data (33). To resolve this problem, proteogenomic analyses are increasingly employed for aiding noncanonical microprotein discovery by integrating genomic, transcriptomic, and proteomic data (34) but remain controversial due to high false-positive rates (35). More recently, the incorporation of translatomics data—particularly Ribo-seq—has been emphasized as an effective strategy to reduce false positives (36, 37). Ribo-seq, a representative translatomics technique, has played a crucial role in noncanonical microprotein discovery (38). Recent studies have combined Ribo-seq with mass spectrometry–based proteomics to systematically explore the translational landscape of non-canonical ORFs across diverse biological contexts. Ouspenskaia *et al.* and Marta *et al.* applied Ribo-seq–informed proteogenomic strategies to investigate the potential of non-canonical ORF-derived products as tumor antigens (39, 40). Ouspenskaia *et al*. identified 3555 translated non-canonical ORFs from MHC-I immunopeptidome mass spectrometry data, whereas Marta *et al*. reported 33 tumor-specific lncRNAs encoding novel cancer antigens shared by more than 10% of hepatocellular carcinoma samples. Using similar proteogenomic strategies, Chothani *et al.* characterized proteomic landscapes across distinct heart regions and embryonic stem cells, identifying 603 small peptides, whereas Duffy *et al*. examined human brain tissues and detected peptides corresponding to 4104 non-canonical ORFs (41, 42). These studies have suggested the broad applicability of translatomics-guided proteogenomic strategies for uncovering translation of non-canonical ORFs across tissues and disease contexts.

Motivated by the recent expansion in lncRNA annotation (NONCODE V6) and more advanced proteomic techniques (6, 43), we explored hepatocellular carcinoma (HCC)-relevant lncPeps using an integrative proteogenomic framework that combines Ribo-seq and proteomic datasets. In contrast to previous work that has largely focused on immunopeptidomics and antigen presentation, our analysis emphasizes global proteome-level detection of lncPeps, an aspect that remains relatively underexplored. Moreover, by leveraging state-of-the-art proteomic datasets, we implemented stringent quality control at both the PSM and peptide assignment levels, followed by an integrated confidence scoring and prioritization framework to systematically evaluate peptide reliability. Using this workflow, we reprocessed high-quality, publicly available human Ribo-seq datasets including five cancerous and two paracancerous liver tissues, as well as five biological samples from primary human hepatocytes and five samples from two liver cancer cell lines. To predict lncORFs, we used GENCODE (version 46) and NONCODE (version 6) as references and three ORF identifiers to generate lncORFs indices (44, 45). The identified lncORFs were translated into a lncORF database in amino acid sequence for lncPep discovery in HCC patients. Biopsy proteomic data from cancerous/paracancerous paired tissues of 268 patients from two published cohorts, were retrieved from published databases and analyzed (6, 43). We identify a total of 104 lncPeps with 46 lncPeps differentially expressed in cancerous tissues (e.g., *HNRNPA1P36* lncORF1), and 13 lncPeps (e.g., *PPIAP79* lncORF1 and *POTEKP* lncORF2) exhibiting significant correlations with patient survival and recurrence prognosis. This study demonstrates the potential of using proteogenomic pipelines to discover functional lncPeps in HCC and possibly other cancers.

## Experimental Procedures

### Ribo-seq Data Collection and Alignment and Quality Control

Ribo-seq datasets were downloaded from the NCBI GEO database (Supplemental Table S1) (46). Trim Galore was used for preprocessing to remove adapter sequences and low-quality reads (47). Human rRNA sequences were downloaded from the Rfam database, and bowtie2 was used to remove rRNA reads (48, 49). The remaining reads were aligned against the human reference genome hg38 using STAR with the following parameters: ‘–outFilterType BySJout –outFilterMismatchNmax 2 –outSAMtype BAM SortedByCoordinate –quantMode TranscriptomeSAM –outFilterMultimapNmax 1 –outFilterMatchNmin 16 –readFilesCommand zcat –outReadsUnmapped None –alignEndsType EndToEnd’ (50). Multi-mappers were discarded.

All Ribo-seq data underwent rigorous quality control, with the methods and criteria detailed below. The metaplot function from RiboCode and the quality function from Ribo-TISH were employed to analyze the ribosome-protected fragments (RPFs) (51, 52). The criteria for quality control required RPF reads to be 27 to 30 nucleotides in length, exhibit triplet periodicity within the coding sequence (CDS) region as determined by both RiboCode and Ribo-TISH and have a P-site offset of 12 nucleotides. Only reads meeting these strict criteria were used for ORF prediction and quantification. After applying these filters, 17 datasets were selected for further analysis (Supplemental Table S1).

BEDTools intersect function was used to analyze the distribution of RPF reads across different RNA features (53). Reads mapped to genomic features were counted using the *featureCount* function from the Subread package and normalized as FPKM values (54, 55).

### Prediction and Assessment of Translational Potentials of Open Reading Frames on lncRNAs

lncRNA annotation files were obtained from GENCODE (Version 46) and NONCODE (Version 6) as reference files for annotating human lncORFs (44, 45). RiboCode, Ribo-TISH, and ribotricer were used to detect actively translating ORFs (51, 52, 56). Across all three tools, the minimum ORF length was set to 18 nucleotides. For translation initiation, ATG, CTG, GTG, and TTG were considered as potential start codons, while TAG, TAA, and TGA were used as stop codons for translational termination. For RiboCode and Ribo-TISH, both the longest strategy and frame-best strategy were used to predict ORFs, with the default *p*-value (Stouffer’s method combined *p*-value <0.05) employed for identifying actively translating ORFs. In the case of ribotricer, the recommended human phase-score cutoff was applied for active ORF prediction, along with a minimum ratio of codons with non-zero reads set to 0.4.

The RPF track for a specific ORF is plotted by RiboCode's *plot_orf_density* function.

To assess the quality of the predicted lncORFs, we did comparative analysis of sequence and translation characteristics for lncORFs, CDS, and randomly intersected lncRNA region controls. Length (nt), ribocode *p*-values, ribotracer phase scores, and read densities (RPF reads per ORF length) were plotted. To generate the lncRNA region control, we randomly sampled 20,000 length-comparable exonic sequences (between first and fourth quartile of lncORFs equivalent to 87–285 nt) from annotated lncRNA transcripts. All sampling procedures were implemented in R.

### Building a Custom Proteogenomics Database

The active ORFs predicted by the three tools were merged and collapsed. For ORFs derived from the same gene, those sharing the same stop codon across different transcript isoforms were combined. The longest isoform was defined as the one harboring the most upstream in-frame start codon. The optimal isoform was defined as the ORF with the lowest *p* value in RiboCode or Ribo-TISH, or the highest phase score in ribotricer.

Based on the Ribo-seq results, we constructed three proteogenomic database candidates with varying levels of stringency. Database one included lncORFs predicted by two or more of the three tools, totaling 9451 lncORFs (RiboCode ∩ Ribo-TISH) ∪ (RiboCode ∩ ribotricer) ∪ (Ribo-TISH ∩ ribotricer). Database two included lncORFs predicted by at least one tool, totaling 33,083 lncORFs (RiboCode ∪ Ribo-TISH ∪ ribotricer). Database three included all candidate ORFs predicted by ORFfinder from transcripts showing RPF signals. In this case, no triplet periodicity filtering was applied, resulting in 288,383 ORFs. These databases reflect different levels of tolerance in defining lncORF pools. Additionally, lncORF annotated by GENCODE (V46) and UniProt (Swiss-Prot) were added (45, 57). To facilitate group FDR estimation, we customized the FASTA headers for the translated lncORF database to distinguish them from canonical proteins.

### Computational Proteomics Analysis

Proteomic data from hepatocellular carcinoma patient biopsies were obtained from the IProX and CPTAC repositories, as reported by Jiang *et al.* and Gao *et al.* (6, 43, 58). The dataset from Jiang *et al.* was generated using label-free quantification, whereas the dataset from Gao *et al.* was based on tandem mass tag (TMT) data 11-plex labeling. Using the custom lncORF indices along with the UniProt (UniProtKB–Swiss-Prot downloaded September 2023, including 41,452 curated human proteins) human protein database, we annotated the datasets following analysis with the FragPipe computational platform (version 22). MSFragger was used for peptide identification, trypsin was selected as the digestion enzyme, allowing up to two missed cleavage sites. Cysteine carbamidomethylation was set as a fixed modification, while N-terminal acetylation and methionine oxidation were considered variable modifications. For the TMT-MS dataset, corresponding TMT modifications were included in the FragPipe database search parameters. The precursor mass tolerance was set to 20 ppm for the initial search and 4.5 ppm for the main search, with a fragment ion mass tolerance of 20 ppm. Known laboratory contaminants were included in the analysis. A reverse-sequence decoy database was generated and used for false discovery rate (FDR) estimation (59).

DIA-NN (version 1.8.2 beta 8, distributed via FragPipe) was utilized to predict MS2 spectra, retention time (RT), and ion mobility (IM), while MSBooster integrated peptide-spectra matching (PSM) features with deep learning-based predictions. Philosopher was used for false discovery rate (FDR) estimation (60, 61). To estimate FDRs for peptides matched to lncORF, we used two strategies: group-specific FDR estimation and the two-pass search (62, 63). In the group-specific FDR estimation, lncORFs and known proteins were analyzed together, but the FDR was calculated separately for each group. In the two-pass search strategy, known proteins were first subjected to FDR estimation. Spectra that did not pass the FDR threshold were then used to search for lncORFs, after which the spectra containing these peptides were subjected to a separate FDR estimation. In both methods, the FDR threshold was set to 1% (64). Group-specific FDR estimation was applied exclusively at the PSM level. Peptide- and ion-level FDRs were then estimated using PSMs that passed a 1% PSM-level FDR filter, with the same cutoff applied at both levels.

All lncORF–derived peptide fragments were mapped against previously annotated proteins in UniProtKB and GENCODE. Peptide fragments that fully mapped to known proteins were excluded. Peptide fragments with a single amino acid mismatch were also excluded as they were considered potential single amino acid polymorphism (SAP) peptides.

The proteomics data viewer (PDV) tool was used as a visualization of the mass spectrometry (65).

### Quality Assessment of Peptides Derived From lncORFs

Assessment was restricted to uniquely assigned peptides to lncORFs. To systematically evaluate the reliability of peptides derived from lncORFs, we performed a comprehensive quality assessment at three complementary levels: spectral support, PSM quality, and peptide assignment accuracy.

Spectral support was evaluated based on the number of spectra supporting each peptide. Spectral counts were obtained from the Jiang *et al*. and Gao *et al*. datasets. In addition, external spectral evidence was assessed using PepQuery, based on 24 independent global proteomic datasets, to quantify peptide-associated spectral counts in external data (66). PSM quality was evaluated using the PSM outputs generated by FragPipe, with PeptideProphet probability serving as the primary confidence metric (67). For each peptide, PSM quality was calculated as the average matching quality across all spectra assigned to that peptide. The reproducibility of peptide-spectrum matches was further assessed using an open-search strategy (68). Based on the open-search results, a Delta score was calculated to quantify reproducibility. Delta score was defined as the difference between the HyperScore of the target peptide and that of the best non-target peptide, normalized by the HyperScore of the target peptide. This score ranges from negative values to 1, with higher values indicating better reproducibility. A Delta score greater than 0 indicates that the target peptide remains the top-ranked match in the open-search analysis. Peptide assignment accuracy was further evaluated using ProteoMapper and miniprot (69, 70). Single–amino acid mismatches, as well as insertion, deletion, or frameshift events, were each counted as one mutation event. For ProteoMapper, peptide sequences were aligned against a neXtProt-annotated PEFF reference database comprising 42,382 protein entries and 21,060,293 single–amino acid variant events. For miniprot, peptide sequences were aligned to the human reference genome (GRCh38).

An overall peptide confidence score was calculated by integrating the abovementioned evaluation metrics using metric-specific thresholds and weighted summation. The relative weights for each evidence category and the confidence scores for all peptides are provided in Supplemental Table S5.

### Quantification of the HCC-lncPeps

The resulting lncPeps spectra were used for quantitative analysis, with the MSstats and MSstatsTMT packages applied for protein abundance estimation and normalization of protein quantification results for label-free (LBF) and tandem mass tag (TMT) data, respectively (71, 72). For LBF data, Tukey's median polish was used for protein abundance estimation, and the equalizeMedians method was applied for normalization. In the case of TMT data, the MSstats method was used for protein abundance estimation, with normalization based on the reference channel. The *removeBatchEffect* function from the limma package was utilized for batch effect removal in TMT quantification results, and principal component analysis (PCA) plots were generated to assess batch effects (73). In all approaches, lncPeps were analyzed alongside canonical proteins.

To address missing values in the expression matrix, we applied two strategies: filtering out samples with missing values and applying minimal imputation, with both approaches included in subsequent analyses.

### Biostatistical Analysis

Logistic regression analysis was performed using *glm* function in the stats package. Cox regression analysis for survival analysis was performed using *coxph* function and *survdiff* function was used for log-rank test from survival package (74, 75). For the log-rank test, patients were stratified by lncPep expression into quartiles. The upper quartile (top 25%) was defined as the high-expression group, and the lower quartile (bottom 25%) as the low-expression group.

For RNA-level analyses, all TCGA data were downloaded from the UCSC Toil RNA-seq Recompute project (76), TPM values were used as normalized gene expression, and missing values were imputed using zero.

### Functional Predictions

GSEA analysis was performed using the *gseGO* function from the clusterProfiler package. For the lncPeps of interest, Pearson correlation coefficients were calculated between their intensities and those of all known proteins. The known proteins were then ranked based on these Pearson correlation coefficients, and GSEA was conducted using *gseGO* with the Gene Ontology (GO) database (77). GO terms with an adjusted *p*-value less than 0.05, corrected using the Benjamini-Hochberg method, were considered significantly enriched.

### Machine Learning Modeling

For the MS-peptide-matched-lncORFs (lncORFs derived from Ribo-seq data that have been detected by proteomic mass spectrometry) versus MS-peptide-unmatched-lncORFs (lncORFs derived from Ribo-seq data but matched peptides were not detected by proteomic mass spectrometry) classification model, the seqinr package and the AAindex database were utilized to extract the physicochemical properties of translated lncORF (78, 79). The caret package was employed for data partitioning and machine learning, with 70% of the data randomly sampled as the training set. A LASSO regression model was used for feature selection: the glmnet package implemented LASSO, the *cv.glmnet* function performed 10-fold cross-validation, and the glmnet function fit a generalized linear model via penalized maximum likelihood. The regularization parameter was determined based on lambda.min (80). The final machine learning model was built using the Random Forest algorithm (81). To address the imbalance in the numbers of MS-matched-lncORFs and MS-unmatched-lncORFs during model training, the *downSample* function from the caret package was used for downsampling, and 10 rounds of downsampling were performed to mitigate random sampling bias (81). Additionally, the *Boruta* function in the Boruta package was used to calculate the variable importance measure (VIM) for the LASSO-filtered features in each downsampling (82). The pROC package was used for plotting the ROC curves (83).

For the cancer tissue classification and prognosis prediction models, the normalized expression of canonical proteins and lncPeps was used as input features. The same feature selection method described above was employed. To enhance the interpretability of the model, we utilized a simpler LASSO regression for modeling, without performing any sub-sampling during training. To compare the model based solely on canonical protein expression with the one incorporating both canonical protein and lncPep expression, a likelihood ratio test was conducted using the *anova* function in the stats package of R.

## Results

### Systematic Assessment of lncORFs in Human Liver Using Coding Potentials

**FIG. 1:**
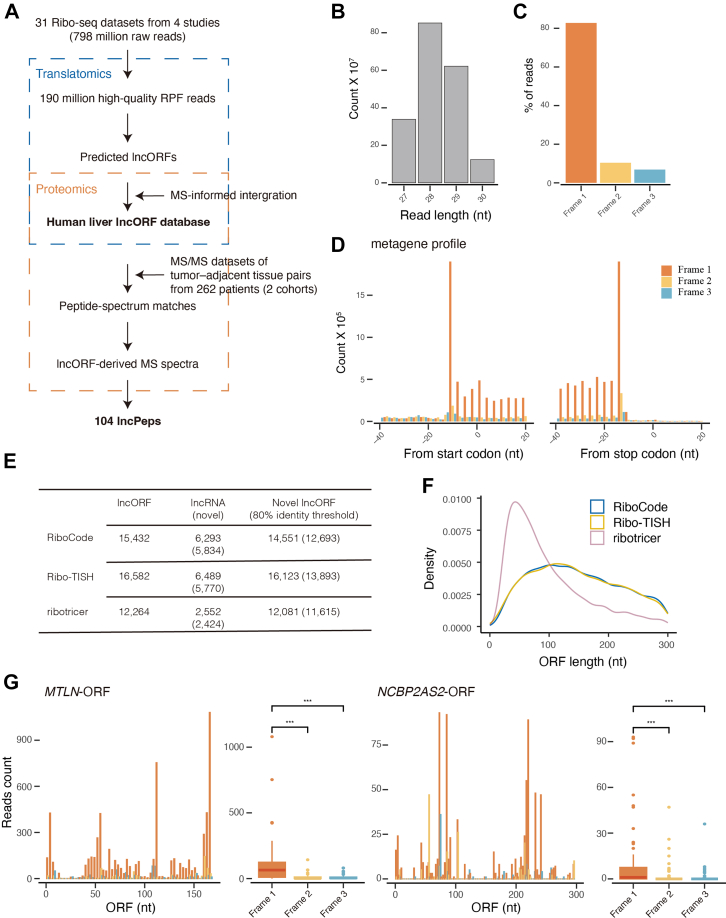
**Identification of three lists oflncORFs using high-quality Ribo-seq datasets and *de novo* ORF identifiers.***A*, flowchart of the study design and discovery analysis. *B*, bar chart demonstrating the overall reads distribution over 27 to 30 nt read lengths across filtered Ribo-seq datasets. *C*, overall frame reads distribution of the filtered Ribo-seq datasets. *D*, metagene profile showing triplet periodicity of P-site distribution in annotated ORFs. *E*, summary of lncORFs and parent lncRNAs predicted by RiboCode, Ribo-TISH, ribotricer *de novo* ORF identifiers. *F*. Kernel Density Estimation (KDE) showing length distribution of identified lncORFs between 15 and 300 nt g. P-site distribution showing triplet periodicity in *MTLN*-ORF and *NCBP2AS2*-ORF (Wilcoxon Rank Sum Test; ∗∗∗, *p* < 0.001).

The proteogenomic discovery strategy employed in this study is illustrated in Figure 1*A*. In brief, the workflow integrates two components: translatomics and proteomics analysis. To systematically investigate lncORFs in human liver tissues and cells, we constructed a liver lncORFs database based on quality-controlled Ribo-seq data derived from relevant biological samples. This database was subsequently used to guide database searches and peptide identification from MS/MS proteomic datasets.

To systematically investigate coding potentials of putative lncORFs in the human liver, we analyzed 31 ribosome profiling sequencing (Ribo-seq) datasets obtained from four independent studies deposited in the GEO database (42, 84, 85, 86). These datasets were derived from human liver cell lines or tissues, including hepatocellular carcinoma (HCC) cell lines, untreated normal human hepatocytes, and cancerous and paracancerous tissues from HCC patients (Supplemental Table S1). In total, these datasets comprised 798 million reads after removal of rRNA-derived reads of which 418 million uniquely mapped reads were retained for downstream analyses. Uniquely mapped reads were predominantly 27 to 30 nucleotides in length (Supplemental Fig. S1*A*). Rigorous quality control was performed on all mapped reads to ensure data reliability (Supplemental Table S1).

After filtering, the processed Ribo-seq dataset comprised 190 million sequencing reads in total, with a length distribution ranging from 27 to 30 nucleotides (Fig. 1*B*). Of the reads, 93.96% read counts were mapped to annotated CDS regions of mRNAs, while the remaining reads were mapped to untranslated regions (UTRs) (4.53%) and ncRNA (1.52%). After length normalization, CDS regions exhibited markedly higher RPF density (4192.5 reads per kilobase, RPK) compared with 5′UTRs (158.6 RPK), 3′UTRs (20.7 RPK), and ncRNAs (5.1 RPK) (Supplemental Fig. S1*B*). Triplet periodicity was confirmed within the annotated CDS, with over 80% of ribosome-protected fragments (RPF) reads aligned to frame 1 (Fig. 1*C*). Additionally, these reads exhibited enrichment at both the CDS start and one codon upstream of stop codons, with a consistent 12-nt offset (Fig. 1*D*). Collectively, these metrics confirm the high quality of the Ribo-seq datasets used in this study (87).

To identify human liver active lncORFs, we extracted candidate ORFs originating from lncRNAs annotated in GENCODE (version 46) and NONCODE (version 6) (44, 45), including GENCODE-annotated lncRNAs, GENCODE-annotated pseudogenes, and NONCODE-annotated lncRNAs. In addition to AUG, we included the near-cognate triplets GUG, CUG, and UUG as alternative start codons, as these codons have been shown to possess translational potential in previous studies (19). We assessed all candidate lncORFs using three Ribo-seq-based active ORF identifiers: RiboCode, Ribo-TISH, and ribotricer. RiboCode and Ribo-TISH are well-established tools for identifying actively translated ORFs, while ribotricer is specifically optimized for detecting translated short ORFs compared with other identifiers (51, 52, 56).

For ORFs derived from the same gene, we first grouped ORFs that shared the same stop codon, which typically represent alternative transcript isoforms and/or ORFs with ambiguous start sites. Within each isoform group, the longest ORF as well as the optimal ORFs was retained (identical in 67% cases). Longest isoform was defined as the isoform harboring the most upstream in-frame start codon. Optimal isoforms were defined as the ORF with the lowest *p*-value in RiboCode or Ribo-TISH, and the isoform with highest phase score in ribotricer. Ribo-TISH identified 16,582 lncORFs from 6489 genes, RiboCode 15,432 lncORFs from 6293 genes, and ribotricer 12,264 lncORFs from 2552 genes (Fig. 1*E*, Supplemental Table S2). The active ORFs identified by these methods were subsequently incorporated into downstream analyses.

We compared the translated lncRNAs identified in this study with those reported in previous studies, including GENCODE Ribo-ORFs, UniProt lncRNA-encoded proteins, and the Chothani *et al.* 2022 study (42, 57, 88). At the gene level, RiboCode and Ribo-TISH showed higher concordance in their identified ORFs, whereas riboticer detected a larger number of active ORFs from fewer lncRNAs (Supplemental Fig. S1*C*). This difference may stem from riboticer’s use of the phase score rather than P-value-based algorithms, a feature previously reported to confer higher sensitivity to shorter ORFs (56). In line with this, the ORFs identified by ribotricer were significantly shorter than those predicted by RiboCode and Ribo-TISH (Fig. 1*F*). We compared our dataset with published resources, including the GENCODE Ribo-ORF dataset and the study by Chothani *et al*. (2022), at both the gene level and the lncORF level. For the lncORF-level comparison, only exact sequence matches were considered. Overlapping lncORFs between this study and previous reports were limited, which may have arisen from a combination of tissue specificity and methodological heterogeneity, including differences in data aggregation strategies and ORF-calling algorithms (Supplemental Fig. S1*C*).

We compared the predicted lncORFs with known CDS regions using three metrics: combined *p*-value (based on the distribution difference of RPF reads across frame 1, frame 2, and frame 3), phase score (ranging from 0 to 1, with higher scores indicating stronger triplet periodicity), and read density (RPF reads per ORF length). The lengths of annotated CDS were significantly larger than the lncORFs (Mean lengths: 1501 nt for CDS, 178 nt for lncORFs). Nonetheless, phase scores and read densities were comparable between lncORFs and CDSs, and significantly higher than lncRNA sequence controls, suggesting similar ribosomal occupancy and translation characteristics. Expectedly, CDS exhibited smaller *p*-values than lncORFs due to their higher read counts and larger numbers of codons contributing to more statistical power in frame-distribution tests (Supplemental Fig. S1*D*).

Experimentally validated HCC-related lncPeps, including KRASIM (99 aa, encoded by *NCBP2AS2*) and MPM (56 aa, encoded by *MTLN*) (26, 27), were successfully predicted by our Ribo-seq analysis by all three identifiers. We further examined the RPF tracks of these two lncPeps. Significant triplet periodicity pattern was observed in the 297 nt and 168 nt ORFs of lncRNA *MTLN* (*p* = 1.09e-15, Stouffer's Method), and lncRNA *NCBP2AS2* (*p* = 6.17e-14, Stouffer's Method) (Fig. 1*G*, Supplemental Fig. S1*E*). These results provide additional validations for the translational potential of the lncORFs.

### Identification of lncPeps From Mass Spectrometry Data

**Fig. 2:**
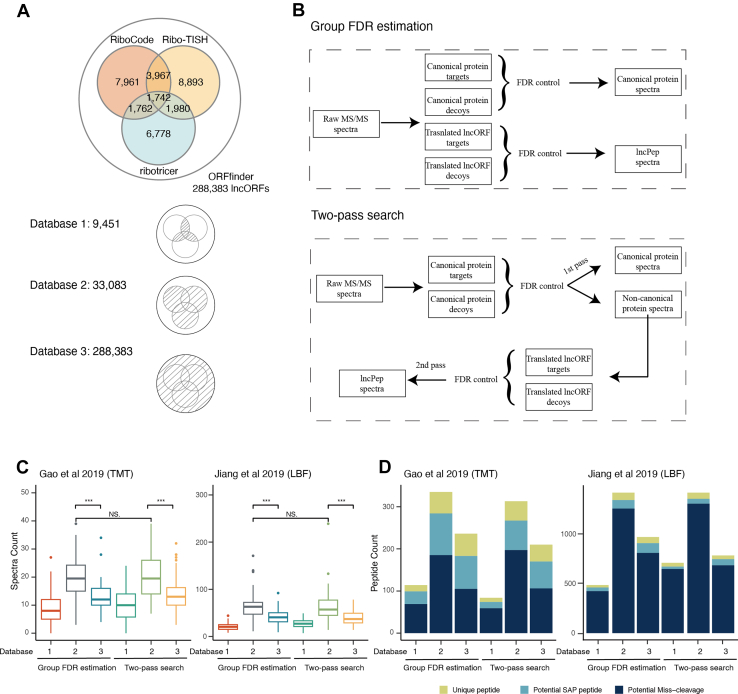
**PSM group FDR control for optimization of the integrated lncORF database.***A*, Venn diagram showing the three overlapping lncORF lists and potential proteogenomic lncORF databases (database 1: lncORFs detected in at least two identifiers; database 2: lncORFs detected in at least one identifier; database 3: all ORFfinder lncORFs). *B*, flowchart of the Group FDR estimation and the Two-pass search as statistical strategies in selecting lncORF databases (FDR threshold 1%). *C*, Box-and-line plots demonstrating the number of putative lncORF-sourced MS/MS spectra identified using each lncORF database: Gao *et al.* (2019) (*left*) and Jiang *et al.* (2019) (*right*). *D*, bar charts showing the number of unique peptides, potential SAP peptides, and potential miss-cleaved peptides detected in Gao *et al.* (2019) (*left*) and Jiang *et al.* (2019) (*right*) datasets.

We constructed three proteogenomics candidate databases from the identified lncORFs. Database one included lncORFs predicted by two or more of the three tools, totaling 9451 lncORFs (RiboCode ∩ Ribo-TISH) ∪ (RiboCode ∩ ribotricer) ∪ (Ribo-TISH∩ ribotricer). Database two included lncORFs predicted by at least one tool, totaling 33,083 lncORFs (RiboCode ∪ Ribo-TISH ∪ ribotricer). Database three included all candidate ORFs from transcripts showing RPF signals. In this case, no triplet periodicity filtering was applied, resulting in 288,383 ORFs (Fig. 2*A*).

lncPeps differ from canonical proteins in length, abundance, and sequence features, which can affect the accuracy of their identification in computational proteomics analyses. Therefore, group-specific false discovery rate (FDR) estimation for lncORF-sourced spectra is critical to ensure reliable lncPep identification (89, 90). We compared the performance of two FDR control strategies: group-specific FDR estimation and the two-pass search method for identifying lncORF-derived spectra (62, 63). The group-specific FDR estimation approach identifies peptides from both canonical proteins and translated lncORFs but applies FDR control separately for each group. In contrast, the two-pass search strategy first performs peptide identification and FDR control on canonical proteins and then evaluates whether the spectra not attributed to canonical proteins may originate from lncPeps (Fig. 2*B*).

To evaluate the performance of the two FDR control methods and the three databases, we analyzed two test datasets using the FragPipe platform. Each test dataset consisted of cancerous and paracancerous tissue samples from five patients, randomly selected from the larger cohorts reported by Jiang *et al.* and Gao *et al* (6, 43).

Database 2, which has the intermediate stringency between database 1 (most stringent) and database 3 (most permissive), outperformed database 1 and database 3 in identifying higher numbers of lncPep-sourced spectra (Fig. 2*C*). Notably, the higher detection sensitivity of database 2 suggests that Ribo-seq-guided proteogenomic approaches impact downstream proteomics analyses. A comparison of Group FDR estimation and two-pass search method found no significant difference in the number of identified lncORF-sourced spectra between the two strategies (Fig. 2*C*).

Peptide fragments fully aligned with canonical proteins but lacking a trypsin cleavage site were considered potential trypsin miscleavages—a common occurrence in trypsin-based proteomics—and were filtered out from subsequent analyses (91). Single amino acid polymorphisms (SAPs) frequently occur in peptides. Therefore, we applied an additional filtering step to spectra that are potentially originating from lncORFs. Fragments aligned to canonical proteins with a single amino acid mismatch were regarded as a potential SAP product. Peptide fragments that could not be aligned to any canonical proteins after allowing one mismatch were considered unique and retained for subsequent lncPep identification and quantification, consistent with thresholds used in previous studies (92). On average, 95% of lncORF-derived peptides in the Jiang 2019 dataset and 83% in the Gao 2019 dataset were mapped to missed cleavage or SAP events and filtered out (Fig. 2*D*).

These pilot results supported database 2 in combination of controlled FDR rate at 1% with group-specific estimation as an effective strategy for downstream lncPep discovery analysis. The detailed workflow is illustrated in Supplemental Figure S2.

### Quality Assessment of Peptides Matched to lncORFs

We analyzed the full proteomic datasets from two previously reported cohorts. In the original studies, Jiang *et al.* profiled 103 hepatocellular carcinoma (HCC) patients at clinically early stages using a label-free (LBF) quantitative proteomics approach, whereas Gao *et al.* reported 159 HCC patients associated with hepatitis B virus infection using tandem mass tag (TMT)–based proteomics (6, 39) (Supplemental Table S3).

Using the pipeline established above, we identified 879 mass spectra corresponding to peptides derived from 43 putative lncORFs in the Jiang *et al*. dataset, and 3538 mass spectra corresponding to 126 putative lncORFs in the Gao *et al*. dataset (Supplemental Table S4). The matched peptide-spectra and their corresponding peptides were subjected to characterization and quality assessment at both the PSM level and the peptide assignment level. At the PSM level, we evaluated spectral support (number of spectra), PSM quality, and PSM reproducibility using open-search analyses (67, 68). At the peptide assignment level, we assessed the accuracy of peptide annotation by mapping peptides to known proteins using ProteoMapper for potential known sequence variants, and by aligning peptides to the genome using miniprot to evaluate whether they could arise from insertions, deletions, or frameshift events (69, 70).

We first compared the PSMs supporting lncORF-derived peptides and canonical ORF–derived peptides. As a reference, canonical ORF–derived peptides were randomly sampled. Several spectral-level metrics were evaluated, including spectral counts, HyperScore, Delta score, and PeptideProphet probability. HyperScore measures the similarity between the observed and the theoretical spectra, with higher values indicating better spectral matches. Delta score represents the difference in HyperScore between the top-ranked peptide and the second-best candidate, with larger values reflecting greater discrimination between competing identifications. PeptideProphet probability provides a confidence score for peptide identification, where higher values indicate greater confidence.

In both the LBF dataset from Jiang *et al*. and the TMT-based dataset from Gao *et al*., peptides derived from canonical ORF were generally supported by a higher number of spectra, consistent with their higher abundance and broader expression. In the LBF dataset, peptides derived from lncORFs exhibited higher HyperScore values than canonical ORF–derived peptides, whereas no such difference was observed in the TMT dataset. Delta score distributions were similar between lncORF-derived and canonical ORF–derived peptides in both datasets, indicating comparable discrimination between the top-ranked and alternative peptide matches. For PeptideProphet probability, lncORF-derived peptides showed a slightly reduced fraction of high-probability identifications relative to canonical ORF–derived peptides, while also exhibiting fewer low-probability matches, resulting in an overall comparable confidence profile (Supplemental Fig. S3). Taken together, these comparisons indicate that peptides derived from lncORFs exhibit PSM quality comparable to canonical ORF.

We further assessed the reproducibility of peptide-spectrum matches in open-search analyses. The open-search strategy allows large mass differences between unmodified peptide sequences and experimentally observed precursor ions, enabling the unbiased detection of post-translational modifications (PTMs) without prior specification. This evaluation is critical for determining whether spectra assigned to peptides derived from lncORFs could instead be explained by canonical ORF–derived peptides carrying unassigned modifications. In the Jiang *et al*. dataset, 21 out of 43 lncORF-derived peptides remained the top-ranked matches in the open-search analyses, as indicated by a Delta score greater than 0, whereas in the Gao *et al*. dataset, 101 out of 126 lncORF-derived peptides retained their top-ranked assignments under the same criterion.

Peptide assignment accuracy was further evaluated using ProteoMapper and miniprot. Single–amino acid mismatches as well as insertion, deletion, or frameshift events were each counted as one mutation event. Among the 144 candidate lncORF-derived peptides, 39 peptides could be mapped to known protein-coding genes when allowing up to two mutation events, and 51 peptides could be mapped when allowing up to three mutation events, based on matches identified by either ProteoMapper or miniprot. No potential insertion or frameshift events were detected, and only one possible deletion event was observed.

We integrated the abovementioned evaluation metrics by applying metric-specific thresholds and summing their corresponding weights to derive an overall confidence score for each peptide. The relative weights assigned to each evidence category, as well as the individual confidence scores for all peptides, are provided in Supplemental Table S5. Based on the resulting confidence scores, peptides were ranked and classified into three confidence tiers. A confidence score of 70 was used to define a high-confidence set (Tier 1), resulting in 36 peptides assigned to this tier. Peptides with scores between 50 and 70 (68 peptides) were classified as Tier 2, while the remaining 40 peptides with lower confidence scores were assigned to Tier 3 (Supplemental Fig. S4).

### Physicochemical Properties Limited the Detection of lncORF by Mass Spectrometry

By integrating the two datasets, we identified a total of 104 lncPep groups originating from 153 lncORFs (Supplemental Table S6). lncPeps that are ambiguous with respect to their ORF start codon or source transcript isoform, or cannot be distinguished based on proteomic data, were grouped into a single lncPep group. We defined high-confidence lncPeps as those supported by multiple unique peptides or containing at least one Tier one peptide. Based on these criteria, 41 of the 104 lncPep groups were classified as high confidence.

**Fig. 3:**
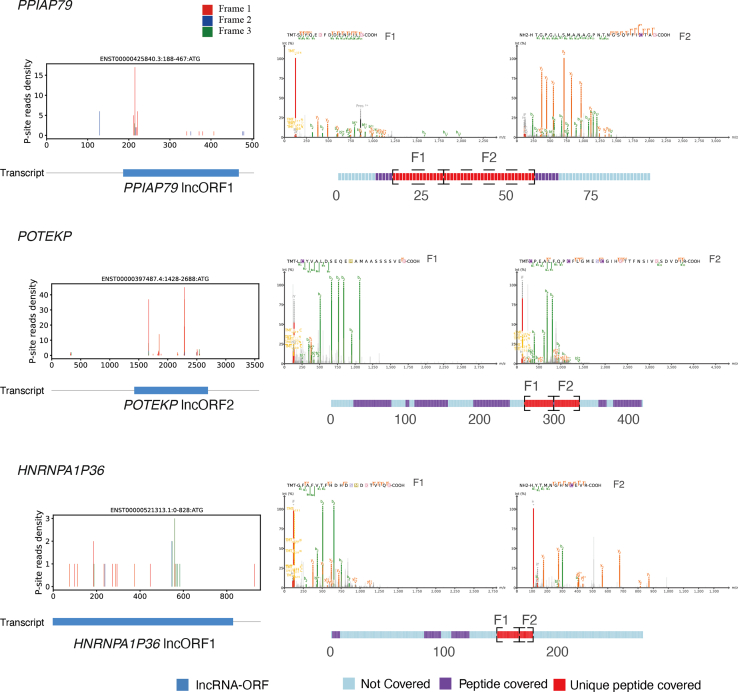
**RPF tracks and matched mass spectra of three lncPep examples.** RPF tracks (*left*) and representative unique peptide MS spectra (*right*) of *PPIAP79* lncORF1 (*top*), *POTEKP* lncORF2 (*middle*), and *HNRNPA1P36* lncORF1 (*bottom*).

From the 104 lncPeps discovered, we present three representative high confidence lncPeps encoded by: *PPIAP79* lncORF1, *POTEKP* lncORF2, and *HNRNPA1P36* lncORF1, along with their corresponding RPF tracks and representative MS spectra (Fig. 3). *PPIAP79* lncORF1 encodes a 92 aa small lncPep supported by two unique Tier 1 peptides. *POTEKP* lncORF2 encodes a 419 aa lncPep that has been cataloged in UniProt (93) and is supported by four unique Tier 3 peptides. *HNRNPA1P36* lncORF1 encodes a lncPep comprising 275 amino acids and is supported by three unique peptides, including one Tier 1 peptide.

**Fig. 4:**
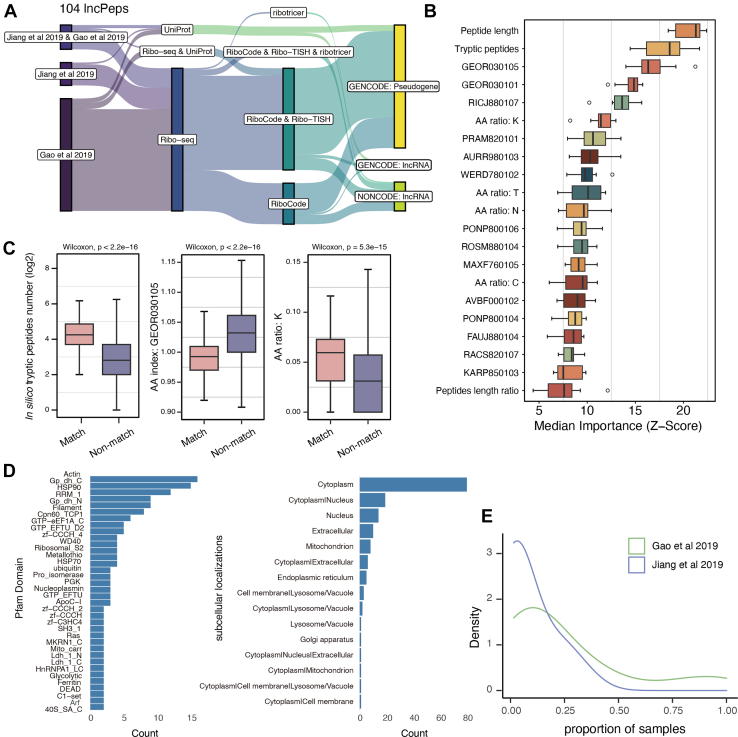
**104lncPeps detected in HCC patient proteomic datasets.***A*, Sankey diagram visualizing traffic flow of the identified lncPeps between publication origin of datasets, databases, ORF identifiers, and biotypes annotated in GENECODE and NONCODE projects. *B*, box plots displaying the “Importance” Z scores of physicochemical properties of translated lncORFs as predictors of MS detectability, using Boruta’s algorithm (higher “Importance” Z-score indicates stronger predictor). *C*, box plots showing *in silico* tryptic peptides number (*left*), GEOR030105 score (*middle*), and lysine ratio (*right*) in MS peptides-matched (match) and MS peptides-unmatched (non-match) lncORF groups (Wilcoxon Rank Sum Test). *D*, predicted Pfam domains and subcellular localizations of the lncPeps. *E*, detection rates of lncPeps across cohort samples.

We traced the sources of these lncPeps based on annotation and found that 84.8% were newly identified in this study. When comparing specific ORF detection algorithms, most lncPep detections were attributed to the overlapping lncORF pools from RiboCode and Ribo-TISH. Some lncPeps were detected exclusively from the RiboCode lncORF pool, while only five ribotricer-predicted ORFs were detected in the mass spectrometry data. In terms of gene origins, most lncPeps were derived from GENCODE-annotated pseudogenes, with some originating from NONCODE-annotated lncRNA genes, and a few from GENCODE-annotated lncRNA genes (Fig. 4*A*).

To understand why a large number of lncORFs (99.5%) were unmatched by mass spectra signals, we investigated the physicochemical properties of MS peptide-matched lncORFs and MS peptide-unmatched lncORFs. The considered properties included 577 features, such as translated ORF length, molecular weight, theoretical isoelectric point, individual amino acid ratios, amino acid family ratios, and AAindex features. After filtering these features, we used LASSO regression for modeling and retained 24 key features that made significant contributions to the distinction.

By analyzing the average importance of variables in each feature, we identified ORF length as the most influential feature. Matched lncORFs were significantly longer than unmatched lncORFs (Fig. 4*B*). To understand whether this is an epiphenomenon of tryptic peptide yield, we incorporated theoretical tryptic peptide count using *in silico* digestion. The contribution of ORF length to MS observability was largely recapitulated by theoretical tryptic peptide count, as reflected by both average feature importance and by ROC (Fig. 4*B*, Supplemental Fig. S5). ORF length divided by the number of theoretical tryptic peptides exhibited substantially lower feature importance. These results suggest that ORF length and tryptic peptide count convey largely redundant information with respect to MS detectability.

Additionally, two AAindex features, GEOR030105 and GEOR030101, showed strong correlations, both associated with the presence of protein domain linkers in microprotein structures (Fig. 4, *B* and *C*) (94). MS peptide-matched lncORFs exhibited a significantly lower propensity for domain linkers. Furthermore, we observed that the AAindex feature RICJ880107, which reflects a tendency for alpha-helix formation, had a significantly lower presence in MS peptide-matched lncORFs (95).

We further applied Random Forest models based on physicochemical features to distinguish matched from unmatched lncORF peptides. Models trained on individual feature categories as well as their combination all showed good discriminative performance, with the combined model achieving the highest accuracy (Supplemental Fig. S5). These results indicate that physicochemical properties differ between MS peptide-matched lncORFs and MS peptide-unmatched lncORFs, supporting a role for such features in MS detectability.

To gain insights into the function of the discovered lncPeps and the cellular pathways they impact, we performed Pfam domain analysis. The most frequently occurring domains were the Actin family, Gp_dh_C, and HSP90 family (Fig. 4*D*). Subcellular localization predicted by DeepLoc2 predominantly localized the lncPeps to the Cytoplasm regions, with smaller fractions assigned to other compartments (e.g., Nucleus, mitochondria, and ER) (Fig. 4*D*).

Next, we calculated the detection rates of these lncPeps across patient samples. In the Jiang *et al.* 2019 dataset, the average detection frequency of lncPeps was 0.085, indicating that on average a lncPep was detected in 8.5% of the samples. In contrast, the Gao *et al.* 2019 dataset, which utilized TMT technology, showed a higher detection rate for lncPeps, with an average frequency of 0.25 (Fig. 4*E*).

### Novel lncPeps Show Potential as Prognostic Biomarkers for Patients With HCC

We performed abundance estimation and biostatistical analysis of these lncPeps to assess their potential clinical utility. PSMs corresponding to unique peptide fragments were used for quantification, with MSstats and MSstatsTMT applied to the LBF and TMT datasets, respectively (71, 72).

We first performed principal component analysis (PCA) on the proteomics expression matrix. In the Jiang *et al.* dataset, PCA of the Merged expression (canonical proteins and lncPep expression) revealed a clear separation between tumor and nontumor tissues, whereas no such separation was observed with lncPep expression alone. Similarly, partial least squares discriminant analysis (PLS-DA), a supervised learning approach, failed to discriminate tumor from non-tumor tissues based solely on lncPep expression (Supplemental Fig. S6, *A* and *B*). This may be attributed to the sparseness of lncPeps expression matrix, which limits their biostatistical significance (Fig. 4*E*). Subsequent analyses demonstrated that tumor and non-tumor tissues were best separable using Merged expression. PCA of lncPep expression captured partial group separation, which was further enhanced by PLS-DA, suggesting that lncPep expression harbors discriminatory information (Supplemental Fig. S6, *C–E*).

To further explore the potential of lncPeps as biomarkers for HCC, we constructed predictive models based on the three types of input data (canonical protein data, lncPep data, or merge of the two) and three distinct tasks comprising tumor classification, survival prognosis, and recurrence prognosis.

**Fig. 5:**
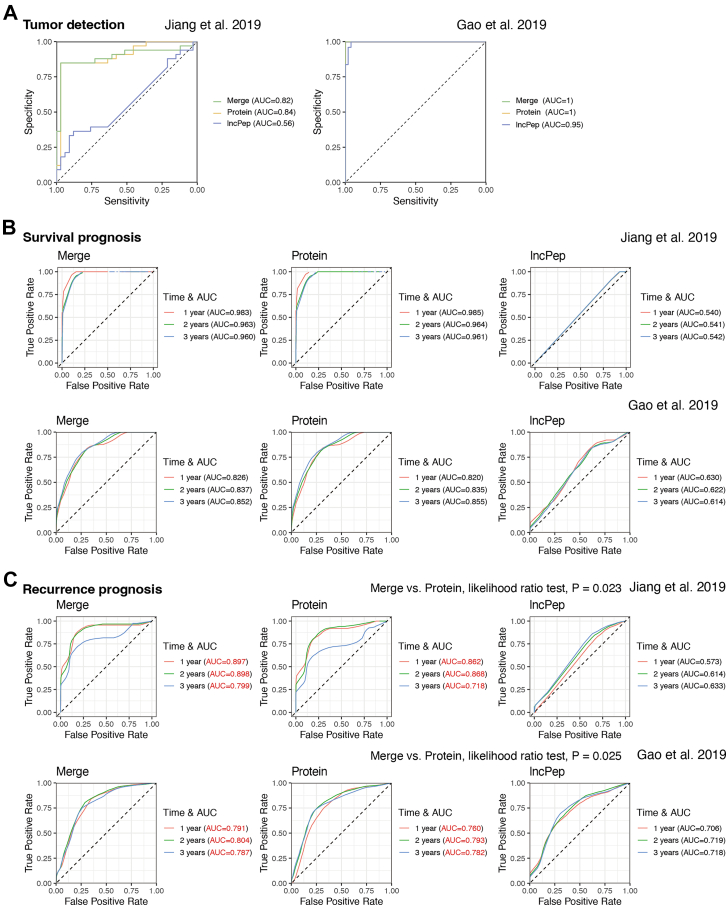
**Improved performance of the HCC recurrence prediction model by combining lncPep expression information.** AUC curves of models based on canonical protein expression, lncPep expression, or Merged for (*A*) tumor detection, (*B*) survival prognosis, and (*C*) recurrence prognosis. Red color denotes AUC values significantly different between Merge and Protein models as detected by Likelihood Ratio Test.

In the tumor tissue classification models using the Jiang *et al.* 2019 dataset, the lncPep-based model performed poorly, and incorporating lncPep expression did not significantly improve the performance of the canonical protein-based model (Fig. 5*A*, lncPep model AUC = 0.56; likelihood ratio test for the merge model vs. the canonical protein model, *p* = 0.68). Similar trends were observed in the Gao *et al.* 2019 dataset, where the lncPep-based model achieved an AUC of 0.95 but remained inferior to the protein-based model, and adding lncPep expression did not significantly improve performance compared with using canonical protein expression alone (Fig. 5*A*, likelihood ratio test for the merge model vs. the canonical protein model, *p* = 1).

For survival prognosis models, incorporating lncPep expression did not improve the performance of the canonical protein-based model in either the Jiang *et al.* 2019 or Gao *et al.* 2019 datasets (Fig. 5*B*; likelihood ratio test for the merge model vs. the canonical protein model, *p* = 0.92 for Jiang *et al.* 2019 and *p* = 0.082 for Gao *et al.* 2019 datasets). In contrast, for recurrence prognosis models, inclusion of lncPep expression significantly enhanced predictive performance in both datasets (Fig. 5*C*; likelihood ratio test for the merge model vs. the canonical protein model, *p* = 0.023 for Jiang *et al.* 2019 and *p* = 0.025 for Gao *et al.* 2019 datasets). These results indicate functional relevance and thus positive contribution of lncPeps to prognostic biomarker development for HCC.

### Individual lncPeps Demonstrate Correlations With Tumors and Patient Prognosis

We then investigated the differences in individual lncPep expression between tumor and non-tumor tissues, as well as the correlation between lncPep expression and patient survival and recurrence rates, using logistic regression and Cox regression analyses. To address missing values in the lncPep expression matrix, we employed two approaches: filtering out samples with missing data or applying minimal value imputation. In addition, we analyzed the TCGA-LIHC (The Cancer Genome Atlas Liver Hepatocellular Carcinoma Collection) dataset to explore the impact of expression changes in the lncRNAs nesting these lncPep (96).

**Fig. 6:**
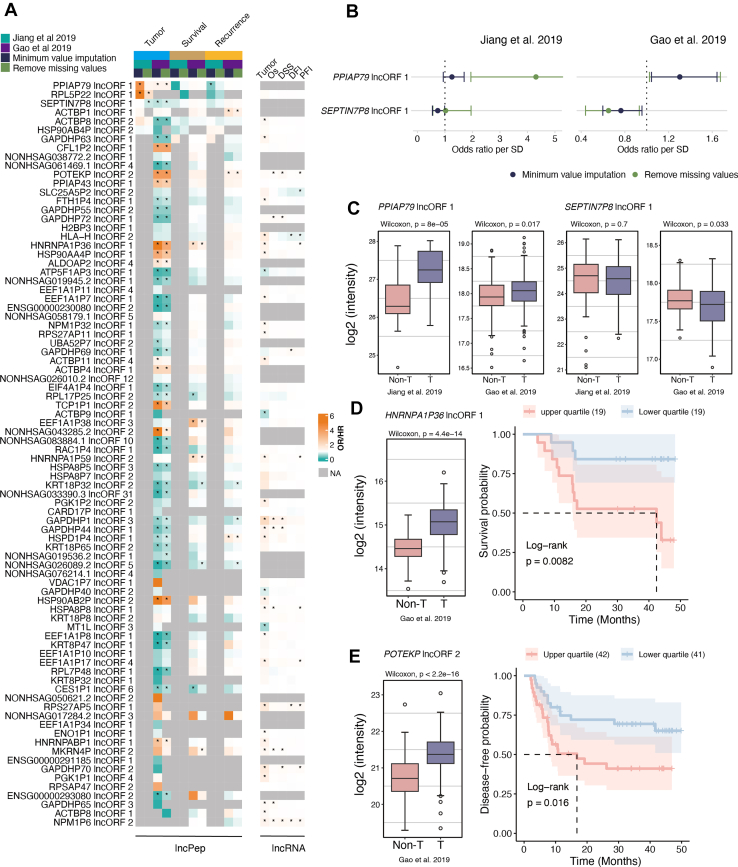
**Newly discoveredlncPeps are associated with tumor, patient survival, and recurrence***A*, heatmap shows the odds ratio (OR) for tumor, hazard ratio (HR) for overall survival prognosis, and HR for recurrence prognosis, mapped against each newly discovered lncPep (*left*) and sourced lncRNA (*right*). Statistical significance was assessed using logistic regression or Cox regression; ∗, *p* < 0.05. *B*, forest plots showing the association between expression of lncPeps (*PPIAP79* lncORF1 and *SEPTIN7P8* lncORF1) and OR for tumor in Jiang *et al.* 2019 and Gao *et al.* 2019. *C*, box plots showing expression of *PPIAP79* lncORF1 and *SEPTIN7P8* lncORF1 in non-tumor tissue (Non-T) and tumor tissue (T). *D*, box plot showing expression of *HNRNPA1P36* lncORF1 in non-tumor and tumor tissues, and Kaplan–Meier (KM) analysis of overall survival probability in patients stratified by *HNRNPA1P36*lncORF1 expression (upper vs. lower quartile). *E*, box plot showing expression of *POTEKP* lncORF2 in Non-tumor and tumor tissues, and KM analysis of recurrence prognosis. Statistical significance was assessed using the Log-rank test. DFI, Disease-Free Interval; DSS, Disease-Specific Survival; OS, Overall Survival; PFI, Progression-Free Interval.

Through this analysis, we identified a series of lncPeps whose expression was associated with cancerous tissue (Fig. 6*A*). We first focused on lncPeps that exhibited consistent effects across both the Jiang *et al.* and Gao *et al.* datasets. lncPep *PPIAP79* lncORF1 demonstrated a significant association with tumor tissues in the Jiang *et al.* dataset (logistic regression, *p* = 0.0013 for remove missing values, *p* = 0.13 for minimum value imputation) and also showed a significant correlation with tumor tissues in the Gao *et al.* dataset using both methods (Fig. 6*B*, logistic regression, *p* = 0.033 for remove missing values, *p* = 0.021 for minimum value imputation). Differential expression analysis revealed that *PPIAP79* lncORF1 was significantly upregulated in cancerous tissues in the Jiang *et al.* dataset (Wilcoxon test, *p* value = 8e-7 for remove missing values, *p* = 0.13 for minimum value imputation) and in both methods using the Gao *et al.* dataset (Fig. 6*B*, Supplemental Fig. S7*A*, Wilcoxon test, *p* value = 0.017 for remove missing values, *p* = 0.01 for minimum value imputation). Similarly, *SEPTIN7P8* lncORF1 showed a significant correlation with non-tumor tissues in the Jiang *et al.* dataset (logistic regression, *p* = 0.94 for remove missing values, *p* = 0.045 for minimum value imputation) and in both methods of the Gao *et al.* dataset (Fig. 6*B*, logistic regression, *p* = 0.023 for remove missing values, *p* = 0.023 for minimum value imputation). Differential expression analysis indicated significant downregulation in paracancerous tissues in the Jiang *et al.* dataset (Wilcoxon test, *p* value = 0.7 for remove missing values, *p* = 0.022 for minimum value imputation) and in the Gao *et al.* dataset (Wilcoxon test, *p* value = 0.033 for remove missing values, *p* = 0.012 for minimum value imputation) (Fig. 6*B*, Supplemental Fig. S7*B*). We were unable to compare RNA expression levels of these pseudogenes in the TCGA-LIHC dataset due to insufficient data (Fig. 6*A*).

We also detected lncPeps with significant associations with patient prognosis. For example, *HNRNPA1P36* lncORF1 was upregulated in tumor tissues and negatively correlated with overall survival (Fig. 6*D*, Supplemental Fig. S7*C*, assessed by both imputation methods), a finding corroborated by Cox regression and log-rank analyses. Similarly, *POTEKP* lncORF2 was significantly upregulated in tumor tissues, and higher expression was associated with poorer disease-free survival (Fig. 6*E*, Supplemental Fig. S7*D*, assessed by both imputation methods), with consistent results observed in Cox regression and log-rank analyses. Analysis of TCGA transcriptomic data showed that, although *POTEKP* RNA levels were not differential between tumor and non-tumor tissues, they were significantly associated with patient prognosis, specifically overall survival (OS), disease-specific survival (DSS), and progression-free interval (PFI) (Supplemental Fig. S8*A*; assessed by both Cox regression and log-rank tests). In contrast, *HNRNPA1P36* RNA was significantly upregulated in tumor tissues, consistent with the protein-level data, and its expression correlated with PFI-associated prognosis (Cox test *p* = 0.03, log-rank test *p* = 0.053) (Supplemental Fig. S8*B*).

We compared lncPeps and canonical proteins for their associations with cancerous tissue, patient survival, and recurrence outcomes after removing samples with missing values. For associations with cancerous tissue, canonical proteins exhibited stronger correlation than lncPeps in both the Jiang *et al*. and Gao *et al*. datasets, a pattern observed for both positive and negative correlations (Supplemental Fig. S9). A similar trend was observed for patient survival outcomes, with canonical proteins showing stronger associations than lncPeps (Supplemental Fig. S9). In contrast, for recurrence outcomes, lncPeps displayed higher correlation than canonical proteins in both datasets. The associations for lncPeps were predominantly negative (Supplemental Fig. S9). We also examined TCGA transcriptomic data to compare the associations of all lncRNAs annotated by GENCODE (v46), highly expressed lncRNAs (top 25% in TCGA-LIHC), and lncRNAs encoding lncPeps with cancerous tissue, patient survival, and recurrence outcomes. For associations with cancerous tissue, highly expressed lncRNAs exhibited stronger correlation than the other lncRNA groups. In contrast, for OS, DSS, disease-free interval (DFI), and PFI, the overall differences in correlation strength among the three lncRNA groups were modest (Supplemental Fig. S10).

In order to explore the biological significance of the three representative lncPeps (*PPIAP79* lncORF1, *POTEKP* lncORF2, and *HNRNPA1P36* lncORF1), canonical proteins were ranked according to their Pearson correlation with lncPep expression and then subjected to GSEA. The results indicated that *PPIAP79* lncORF1 expression was positively correlated with proteins involved in mitochondrial gene expression and mitochondrial translation. *POTEKP* lncORF2 expression showed significant positive correlations with proteins related to the humoral immune response, complement activation, and other immune-related processes. In contrast, *HNRNPA1P36* lncORF1 expression was positively correlated with proteins involved in mRNA processing, chromatin organization, and related cellular processes (Supplemental Fig. S11).

Collectively, these results demonstrate that specific lncPeps expressions are associated with HCC tumors or patient prognosis, suggesting that lncPeps may have functional roles in HCC tumorigenesis and progression, or alternatively, represent byproducts of these processes.

## Discussion

Proteogenomics, defined as the integration of proteomics with genomics and/or transcriptomics, has facilitated the annotation of an increasing number of lncPeps. However, this approach still faces several limitations. Specifically, the sequence content of proteogenomic databases serves as an informed prior about sample composition, and incorrect prior assumptions can negatively impact peptide and protein identification. In practice, this means that a larger reference database search space can reduce identification sensitivity at a given false discovery rate (97, 98). Inspired by recent advances in integrative translatomics–proteomics strategies, we constructed an analysis workflow that incorporates tissue-specific ribosome profiling data into proteomic interpretation. Briefly, the workflow begins with the prediction of lncORFs using Ribo-seq data from liver and hepatocyte cell lines to construct a tailored proteogenomic database. MS-based proteomics data are then mapped to this database, and novel lncPeps are identified following rigorous filtering steps. This approach enables both stringent peptide detection and quantitative comparisons across patients. Applying this workflow, we identified a total of 104 lncPep groups from the cancerous and paracancerous tissues of two independent HCC cohorts.

To optimize the computational proteomics pipeline for maximizing the detection of lncPep-derived spectra under the FDR control level (1%), we evaluated two group FDR control methods and three different tolerance levels of the lncORF database. Our results showed that, although the two-pass search method identified substantially more spectra matching lncPeps compared to group FDR estimation, the sensitivity for lncPep-derived spectra was comparable between the two methods. For different levels of database tolerance, we found that the moderately permissive database two exhibited higher sensitivity. This observation is consistent with the previous notion that a reference database that is too small may fail to match spectra, whereas an excessively large database also reduces identification sensitivity. Optimizing Ribo-seq–derived prior information played a key role in improving the accurate identification of lncPeps.

Of note, among the lncPeps identified in this study, the majority originated from pseudogenes rather than intergenic or antisense lncRNAs. This observation is consistent with previous reports in human epidermal carcinoma and B-lymphoblast cell lines (39, 99). Pseudogenes are duplicated copies of protein-coding genes that have lost the ability to produce functional protein products identical to their parental genes, while retaining a high degree of sequence similarity. Based on this property, pseudogenes have long been hypothesized to exert regulatory functions through the transcription of lncRNAs that modulate the expression of their parental genes (100). Notably, pseudogenes often exhibit cancer-specific expression patterns (101), and in some cases their transcripts are more abundant in tumor tissues than in normal counterparts (102, 103). In contrast, previous immunopeptidome datasets have identified a substantially greater number of lncPeps derived from intergenic or antisense lncRNAs than from pseudogenes (39). This distinction may reflect that lncPeps encoded by lncRNAs of different genomic origins fulfill distinct biological roles.

Previous studies by Yang *et al*. demonstrated that ORF length is the most important determinant of microprotein detectability by mass spectrometry, based on logistic regression analyses of both lncRNA- and mRNA-derived microproteins (104). Consistent with these findings, our analysis likewise identified ORF length as the most influential feature associated with MS detectability of lncPep. We found that the impact of ORF length on MS observability was largely recapitulated by the number of theoretical tryptic peptides generated from the ORFs, indicating that length dependence could be primarily driven by differences in proteolytic fragment generation. In addition, we found that these MS-microproteins are less likely to constitute protein linker regions, which are typically enriched in intrinsically disordered residues (105). α-helix–related AAindex scores also exhibited a negative association with MS detectability. Although α-helical structure is commonly associated with protein stability, increased α-helical content may impede proteolytic accessibility and peptide release, thereby reducing MS detectability (106). Together, these results underscore physicochemical properties as key determinants of lncPep detectability by mass spectrometry and suggest that limited tryptic accessibility represents a major source of bias against short ORFs. This bias likely constrains the proteomic detection of the predominantly short ORFs predicted by ribotricer, thereby providing a plausible explanation for both the low recovery of ribotricer-derived lncORFs in trypsin-based proteomics and the more frequent identification of microproteins in immunopeptidomics datasets (92).

Of the three lncPeps we focused on, *POTEKP* lncORF2 has been cataloged in UniProt, whereas the other two were discovered in this study. Both *POTEKP* lncORF2 and *HNRNPA1P36* lncORF1 were differentially expressed between tumor and non-tumor pairs and show significant associations with patient prognosis. Interestingly, the correlation with prognosis was also observed for RNA expression in the TGCA RNA-seq data, thus the noncanonical protein coding may provide an alternative mechanism in addition to competing endogenous RNA (ceRNA) to explain the correlation between cancer and RNA expression. Previous reports have suggested a *POTEKP*-encoded product showing sequence similarity to beta-actin, which has implications in hepatocellular carcinoma, consistent with our findings (93). In this study, the observed association between *POTEKP* lncORF2 and patient prognosis may reflect a potential role of actin cytoskeleton components in HCC progression. Nevertheless, our understanding of the *POTEKP* remains limited.

We noticed that none of the three lncORFs demonstrated strong RPF tracks in the Ribo-seq analysis. This phenomenon may be due to the fact that they are all derived from pseudogenes that have similar sequences to the corresponding protein-coding genes, causing mapping ambiguity of the RPF reads.

The results collectively affirm the discovery potential of the proteogenomic pipeline constructed in this study. Although our understanding of these lncPeps remains highly limited, emerging evidence suggests that they play important biological roles in human physiological functions and diseases. Our work warrants future investigations of lncPeps in more cancer types to help better characterize tumor heterogeneity and expand the pool of biomarkers, and ultimately benefit patients.

## Data Availability

This study utilized publicly accessible datasets, with the Ribo-seq data listed in Supplemental Table S1, and protein mass spectrometry data obtained from the iProX and CPTAC projects listed in Supplemental Table S3.

The R scripts for summarizing the results and generating the figures are available at https://github.com/lbwfff/lncRNA_derived-peptide_in_HCC and a custom R package developed to ensure analysis reproducibility is available at https://github.com/lbwfff/TNSMD.

## Supplemental Data

This article contains supplemental data.

## Conflict of interest

The authors declare that they have no conflicts of interest with the contents of this article.
