# Supplementary material for: A Proteogenomic Approach to Discover Novel lncRNA-Derived Microproteins and Their Potential Clinical Utility in Hepatocellular Carcinoma
Source: Mol Cell Proteomics. 2026 May 14;25(6):101584. doi: 10.1016/j.mcpro.2026.101584 (PMC13272525; doi:10.1016/j.mcpro.2026.101584)
Supplement: Supplemental Figures [file mmc7.pdf]

A proteogenomic approach to discover novel lncRNA-derived microproteins and their potential clinical utility in hepatocellular carcinoma

Bingwu Li[1],Kandarp Joshi[2,3],Dan Ohtan Wang[1,#]

[1] Biology Program, Division of Science, New York University Abu Dhabi, Abu Dhabi, UAE

[2] Department of Oncology, Sidney Kimmel Comprehensive Cancer Center, School of Medicine, Johns Hopkins University, 1650 Orleans St, Baltimore, MD, 21231, USA

[3] Johns Hopkins All Children's Hospital, 600 5th St. South, St. Petersburg, FL, 33701, USA

Dan Ohtan Wang, Ph.D. Division of Science, C1-031 New York University Abu Dhabi, Al Saadiyat, Abu Dhabi, UAE.

Tel: +971-2-628-5293, Email: ohtan.wang@nyu.edu

List of supplementary tables:

Supplementary Table 1. Information on the Ribo-seq data sources, reads stats, RPF length distribution, and QC.

Supplementary Table 2. lncORFs identifiers using RiboCode, Ribo-TISH, ribotricer.

Supplementary Table 3. Information on the proteomics datasets used in this study.

Supplementary Table 4. PSM information on detected lncORF-derived peptides in Jiang 2019, Gao 2019, and peptides with potential protein source after allowing mismatches based on ProteoMapper and minprot.

Supplementary Table 5. 145 detected lncORF-derived peptides with quality scores and confidence level.

Supplementary Table 6. Information on the 104 lncPeps.

List of supplementary figures:

Figure S1. Supplementary figure for Figure 1

Figure S2. Supplementary figure for Figure 1
Figure S3. Supplementary figure for Figure 3
Figure S4. Supplementary figure for Figure 3
Figure S5. Supplementary figure for Figure 4
Figure S6. Supplementary figure for Figure 5
Figure S7. Supplementary figure for Figure 6
Figure S8. Supplementary figure for Figure 6
Figure S9. Supplementary figure for Figure 6
Figure S10. Supplementary figure for Figure 6
Figure S11. Supplementary figure for Figure 6

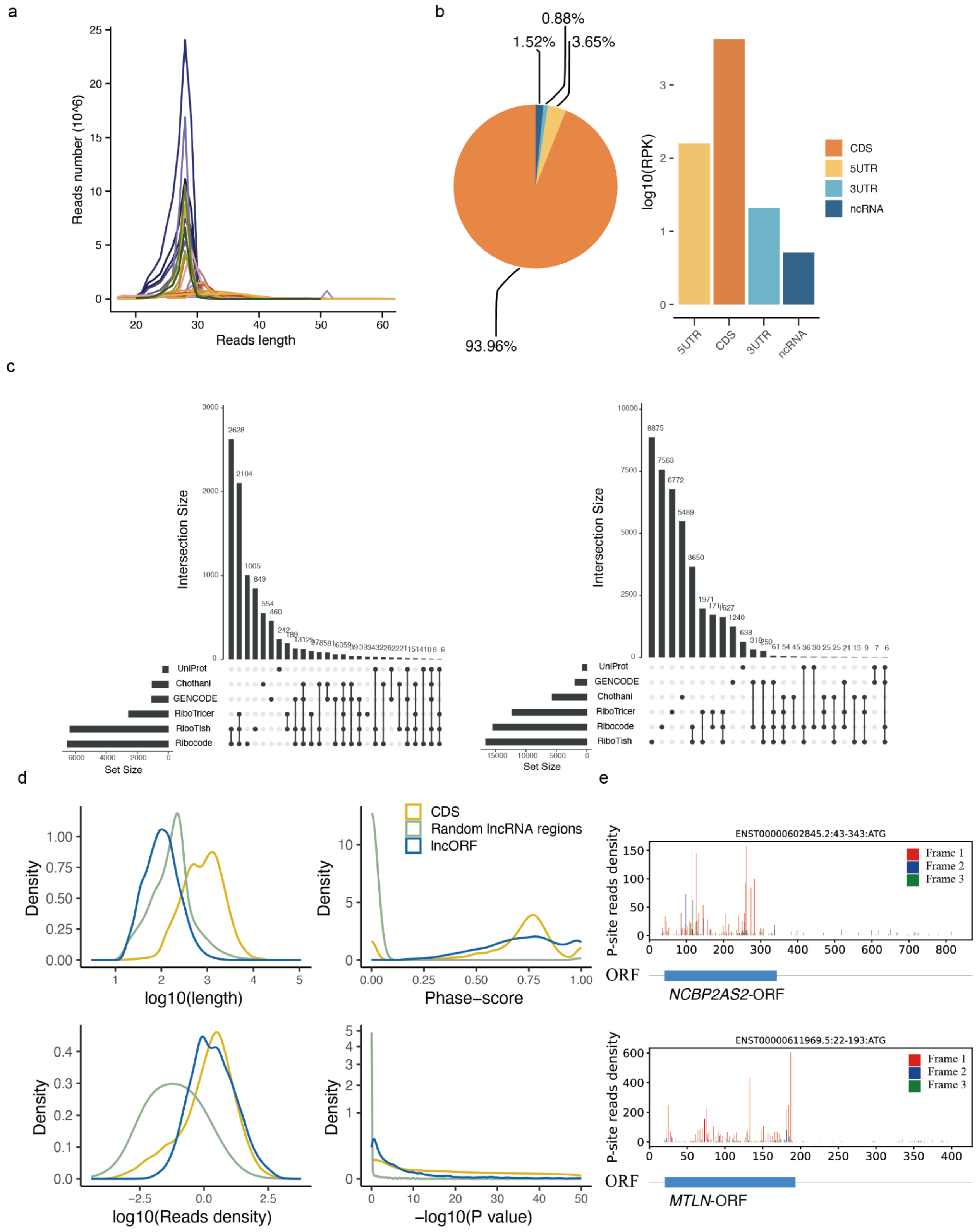

Figure S1: Supplementary figure for Figure 1

a. Read length distributions of rRNA-depleted Ribo-seq reads are shown, with each colored line corresponding to an individual sample. b. Distribution of Ribo-seq reads across transcriptomic features, including coding sequences (CDS), untranslated regions (5UTR, 3UTR), and non-coding RNAs (ncRNAs). The pie chart shows the distribution based on raw read counts; the bar graph presents length-normalized RPF density (reads per kilobase, RPK) across different features. c. Overlap of identified lncRNA genes (left) and lncRNA-ORFs (right) detected by three tools in this study, with annotations from GENCODE, Chothani et al. (2022), and the UniProt database. Only bars representing counts greater than 5 are shown. d. Distribution of ORF length, phase scores, read densities and P-values (Stouffer's method) for predicted lncRNA-ORFs and known coding sequences (CDSs) and random lncRNA regions. e. Distribution of RPF reads across transcripts of *NCBP2AS2* and *MTLN*.

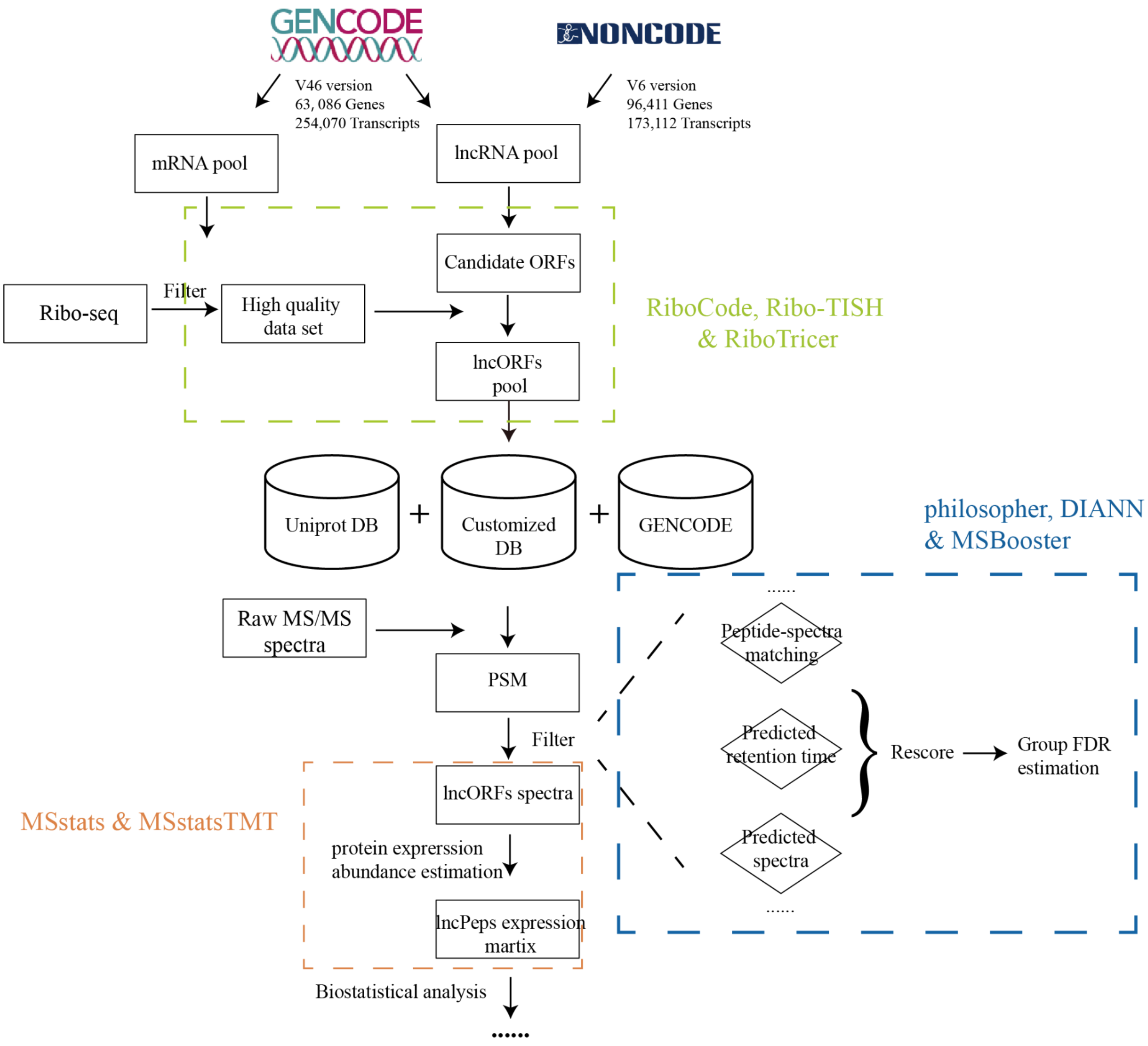

Figure S2: Supplementary figure for Figure 1.

Workflow of lncPep discovery in this study. High-quality Ribo-seq datasets were curated and annotated using GENCODE and NONCODE references. Predicted lncORFs were used to guide proteomic analysis, enabling the identification of high-confidence lncPeps from HCC patient biopsy samples.

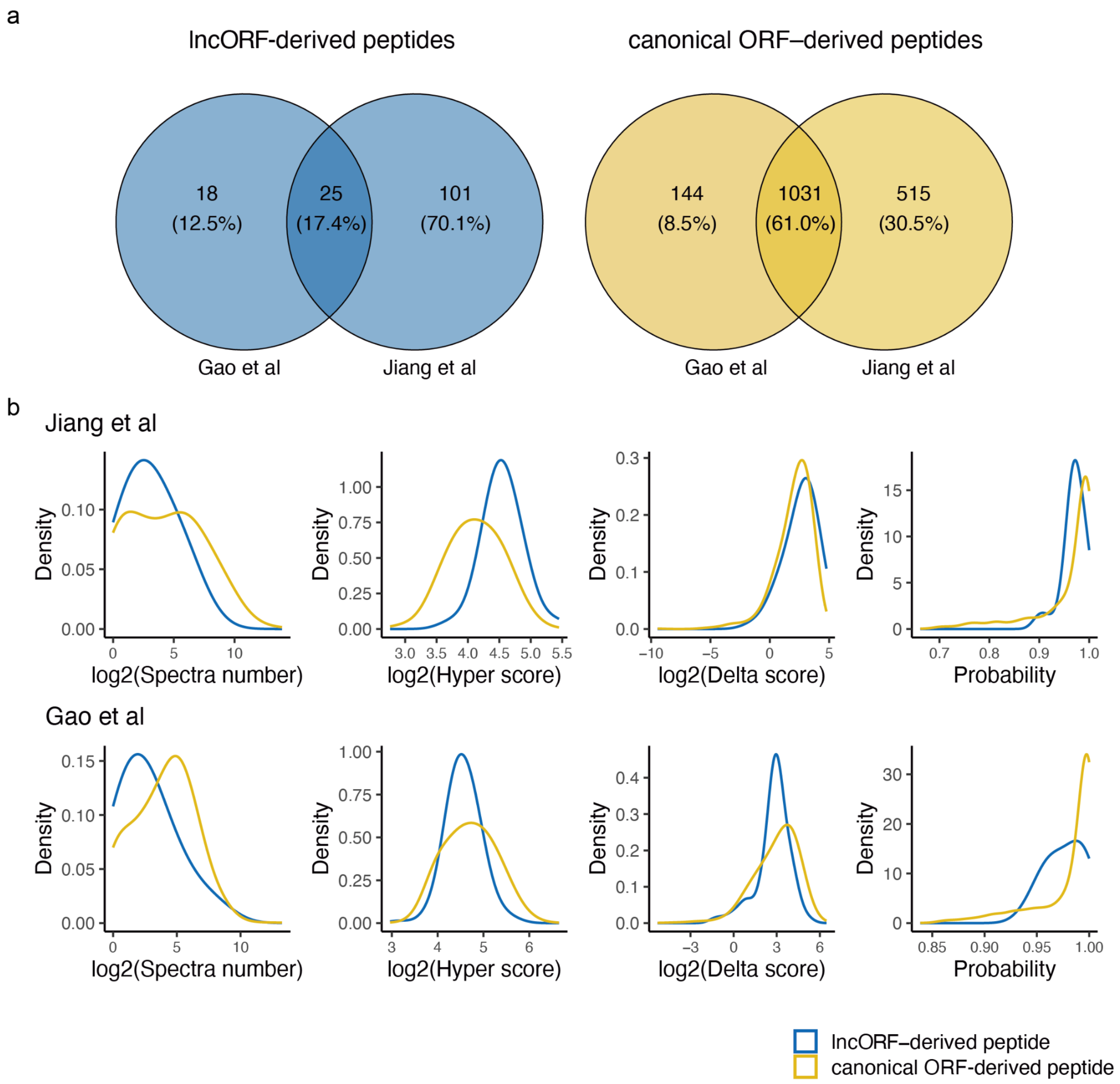

Figure S3 : Supplementary figure for Figure 3

a. Venn diagram showing overlapping lncORF-derived peptides (left) and canonical ORF–derived peptides (right) in Gao et al. (2019) and Jiang et al. (2019) datasets. b. Distributions of spectral counts, HyperScore, Delta score, and PeptideProphet probability for each peptide in Jiang et al. (2019) (top) and Gao et al. (2019) (bottom) datasets.

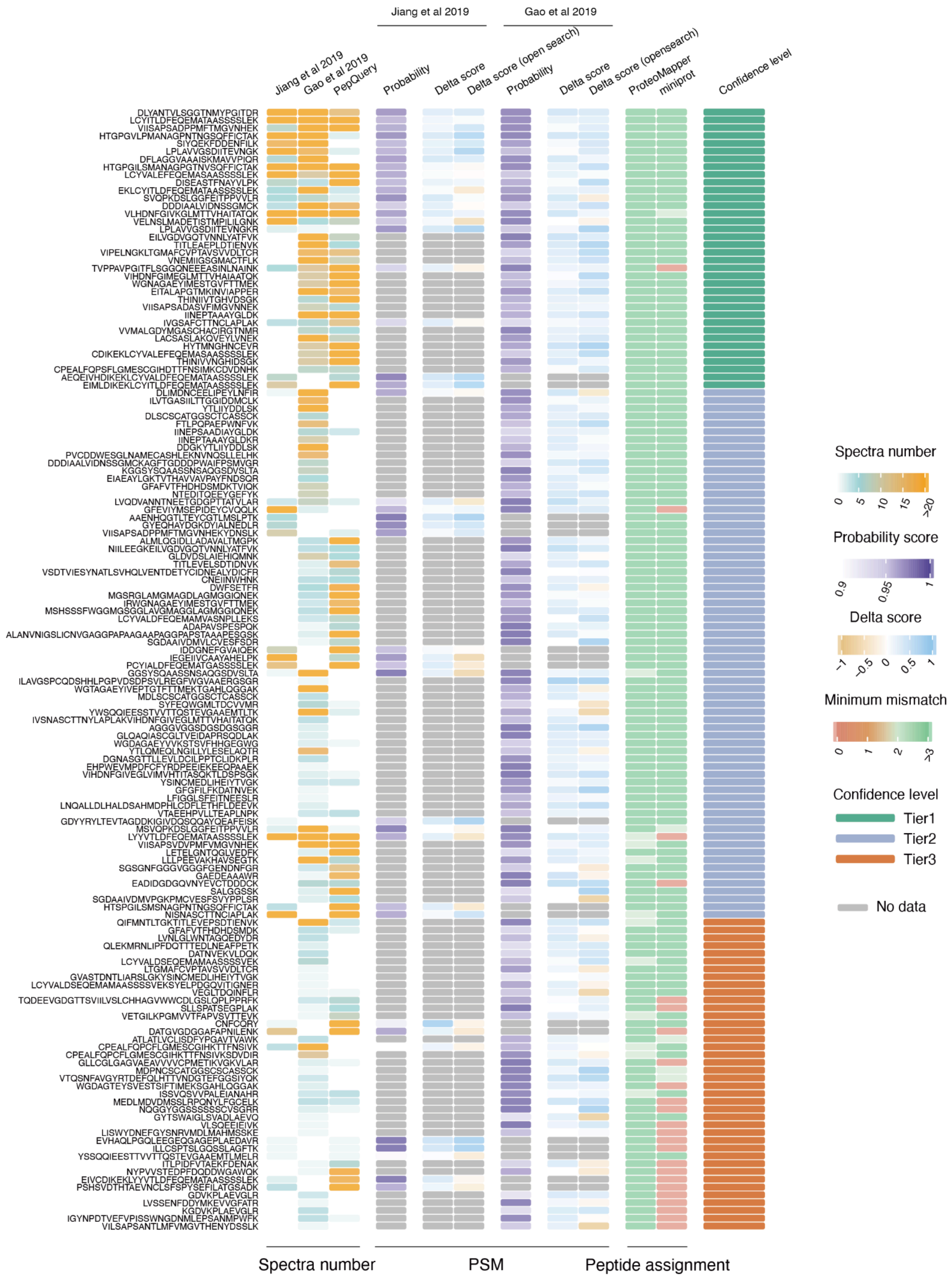
Jiang et al 2019
Gao et al 2019
Jiang et al 2019
Gao et al 2019
PepQuery
Probability
Delta score
Delta score (open search)
Probability
Delta score
Delta score (opensearch)
ProteoMapper
miniprot
Confidence level
Spectra number
0
5
10
15
>20
Probability score
0.9
0.95
1
Delta score
-1
-0.5
0
0.5
1
Minimum mismatch
0
1
2
>3
Confidence level
Tier1
Tier2
Tier3
No data
Spectra number
PSM
Peptide assignment

Figure S4: Supplementary figure for Figure 3

Heatmaps showing quality metrics of lncORF-derived peptides, including spectral number, PSM-level probability score, delta score, and three integrated confidence levels. Spectral numbers include both counts derived from the Jiang 2019 and Gao 2019 datasets and independent external spectral evidence quantified using PepQuery. PeptideProphet probability of peptide-spectrum matches ranges from 0 to 1, with higher values indicating greater confidence. Delta score quantifies the relative separation between the best and the second-best peptide-spectrum matches by measuring the normalized difference in HyperScore; higher values indicate stronger discrimination between competing peptide assignments, and values greater than 0 indicate that the peptide represents the top-ranked match. ProteoMapper and miniprot represent the minimum number of mismatches or mutation events required to reconcile a peptide with known protein-coding sequences or genomic alignments, respectively, with larger values indicating lower likelihood of alternative explanations. The integrated confidence level summarizes these metrics using a weighted scoring framework described in the Methods and is defined as three tiers, with Tier 1 representing the highest confidence.

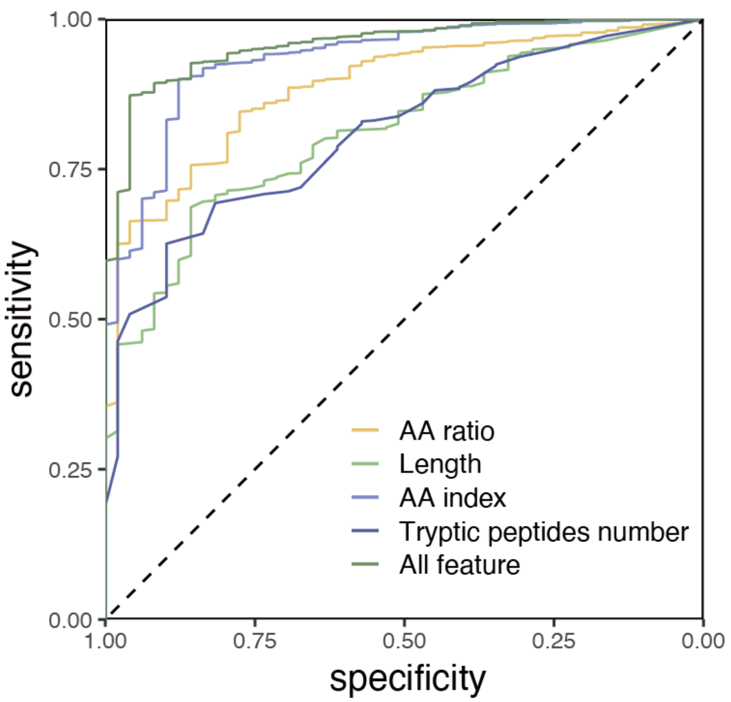

Figure S5: Supplementary figure for Figure 4

Prediction performance of machine learning models trained on amino acid composition, translated lncORF length, AAindex features, *in silico* tryptic peptides number, or all features, evaluated on MS peptides-matched (match) and MS peptides-unmatched (non-match) lncORF groups in the test set (30% lncORFs).

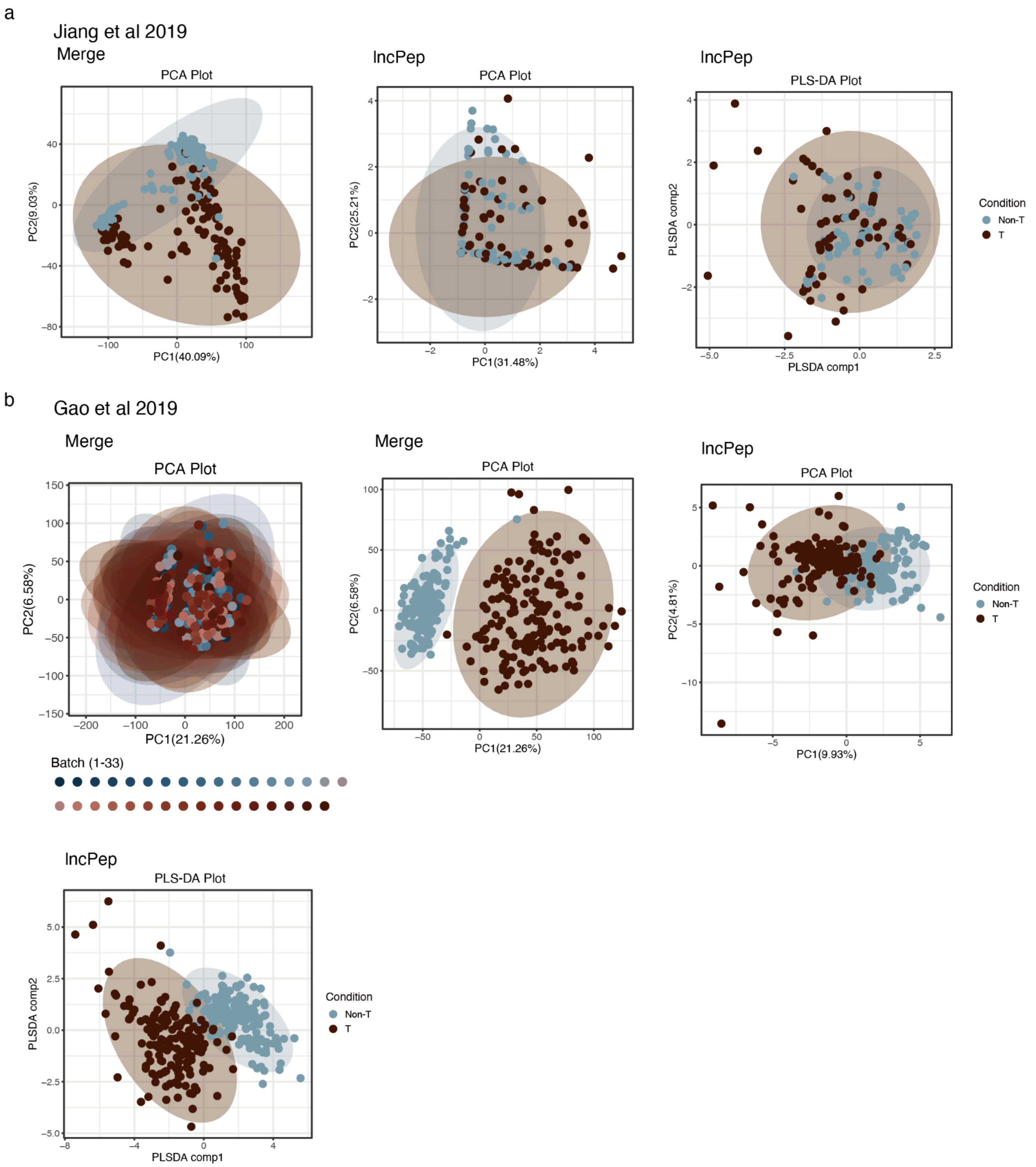

Figure S6: Supplementary figure for Figure 5

a. Principal component analysis (PCA) of the Jiang et al., 2019; Merge, lncPeps, and Partial least squares discriminant analysis (PLS-DA) of lncPeps. b. PCA of the Gao et al., 2019 Merge (grouped by TMT batch 1-33), Merge, lncPeps, and PLS-DA of lncPep. Non-T: Non-tumor and T: tumor tissues.

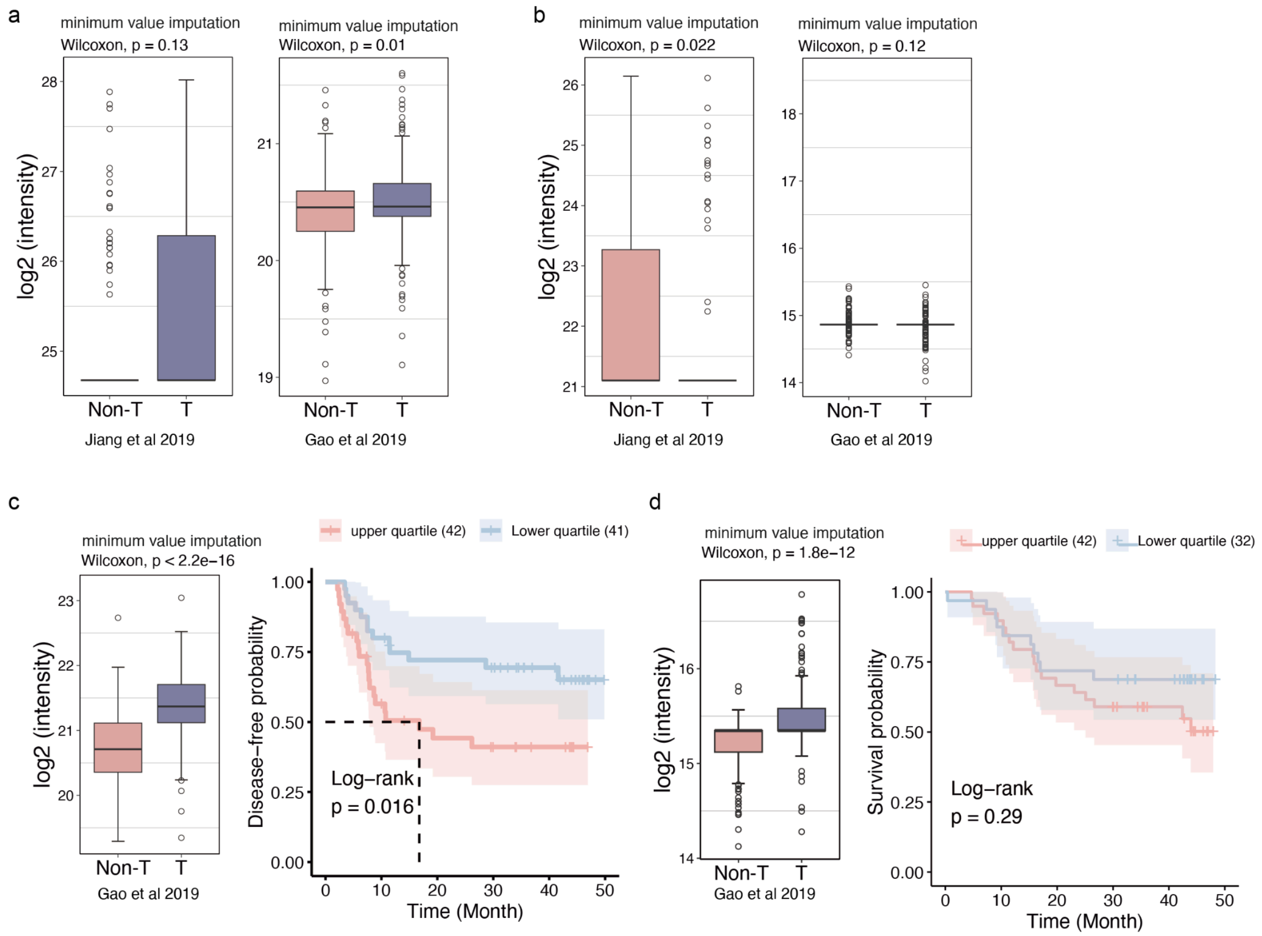

Figure S7: Supplementary figure for Figure 6

a–b. Box plots showing expression of *PPIAP79* lncORF1 (a) and *SEPTIN7P8* lncORF1 (b) in the Jiang et al. 2019 and Gao et al. 2019 datasets. Minimum value imputation was applied. Non-T: non-tumor, T: tumor tissues. c. Box plot showing expression of *POTEKP* lncORF2 in the Gao et al. 2019 dataset, and Kaplan–Meier (KM) analysis of survival probability for patients stratified by *POTEKP* lncORF2 expression (upper vs. lower quartile). Minimum value imputation was applied. d. Box plot showing expression of *HNRNPA1P36* lncORF1 in the Gao et al. 2019 dataset, and Kaplan–Meier (KM) analysis of survival probability for patients stratified by *HNRNPA1P36* lncORF1 expression (upper vs. lower quartile). Minimum value imputation was applied.

a

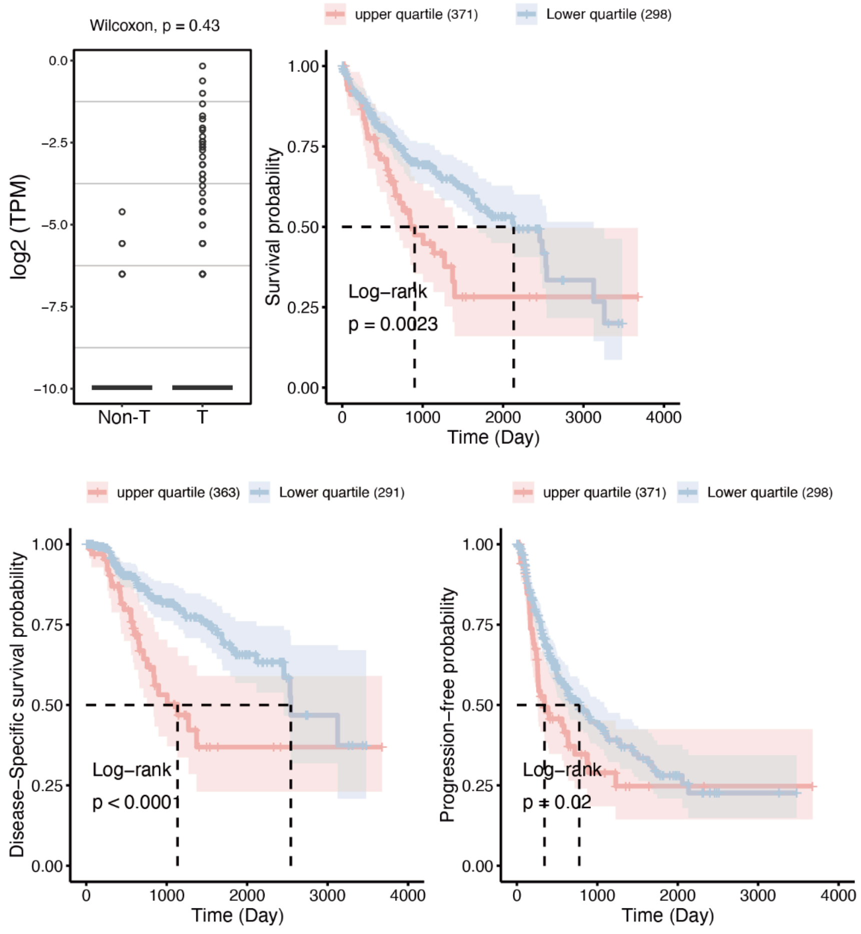

b

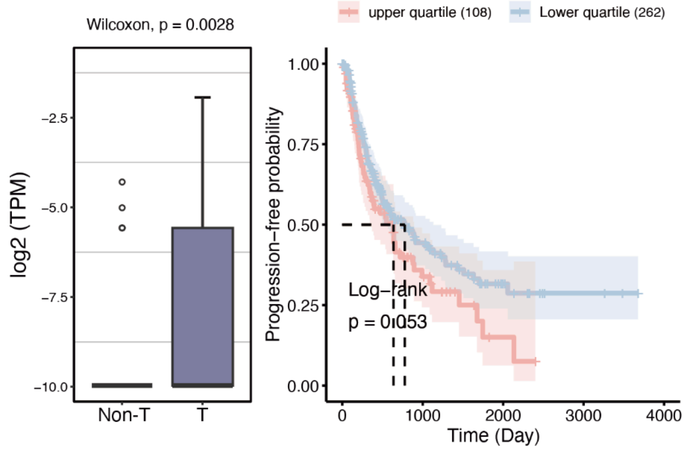

Figure S8: Supplementary figure for Figure 6

a. Box plot showing POTEKP RNA expression in non-tumor (Non-T) and tumor (T) samples from the TCGA dataset, and Kaplan–Meier (KM) analysis of overall survival (OS),

disease-specific survival (DSS), and progression-free interval (PFI) for patients stratified by POTEKP expression (upper vs. lower quartile). b. Box plot showing HNRNPA1P36 RNA expression in the TCGA dataset, and KM analysis of progression-free interval (PFI) for patients stratified by HNRNPA1P36 expression (upper vs. lower quartile).

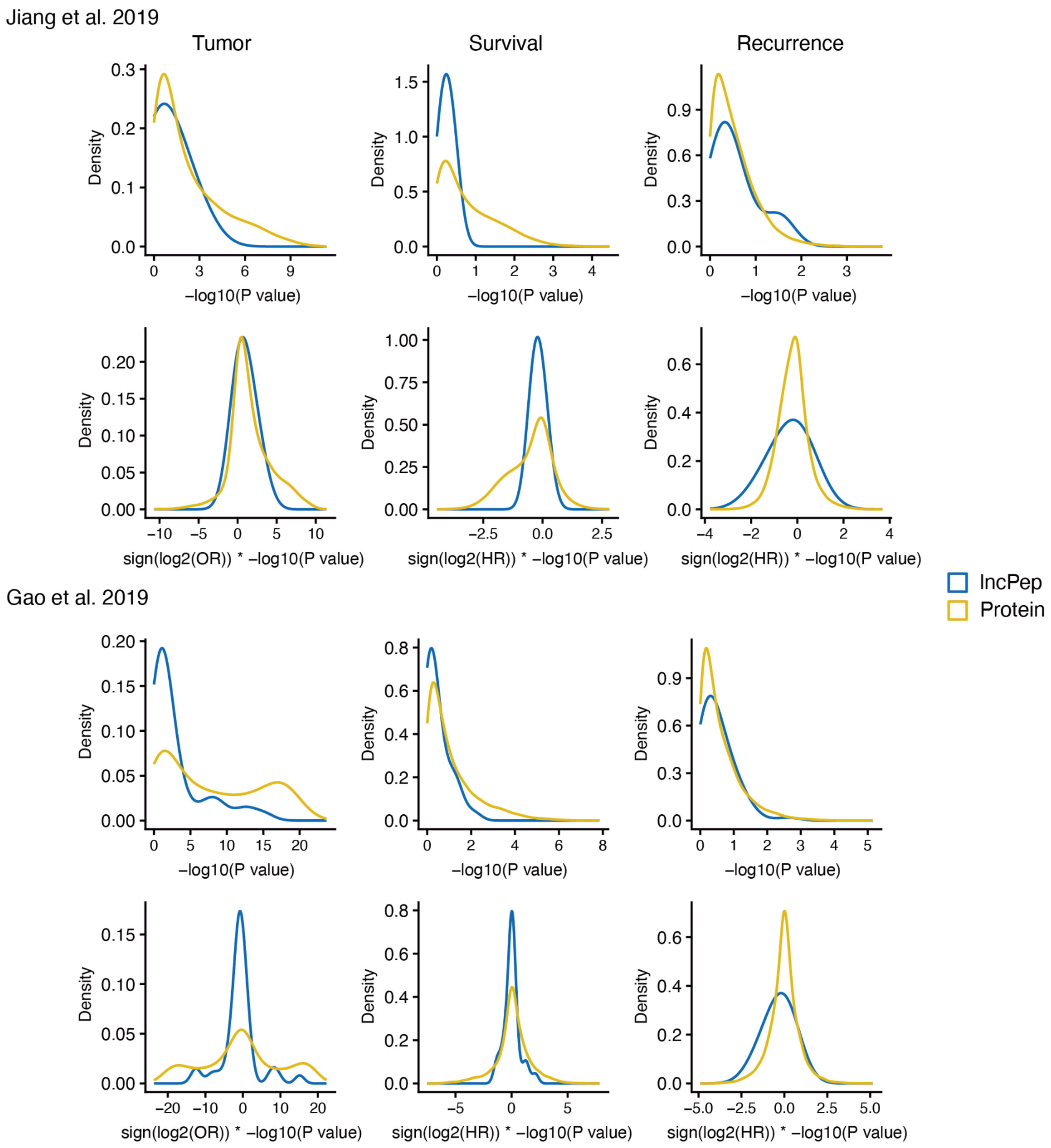

Figure S9: Supplementary figure for Figure 6

Density plots showing the distributions of odds ratios (ORs) for cancerous tissue, hazard ratios (HRs) for overall survival, and HRs for recurrence-free survival for lncPeps and canonical proteins in the Jiang et al. (2019) (top) and Gao et al. (2019) (bottom) datasets.

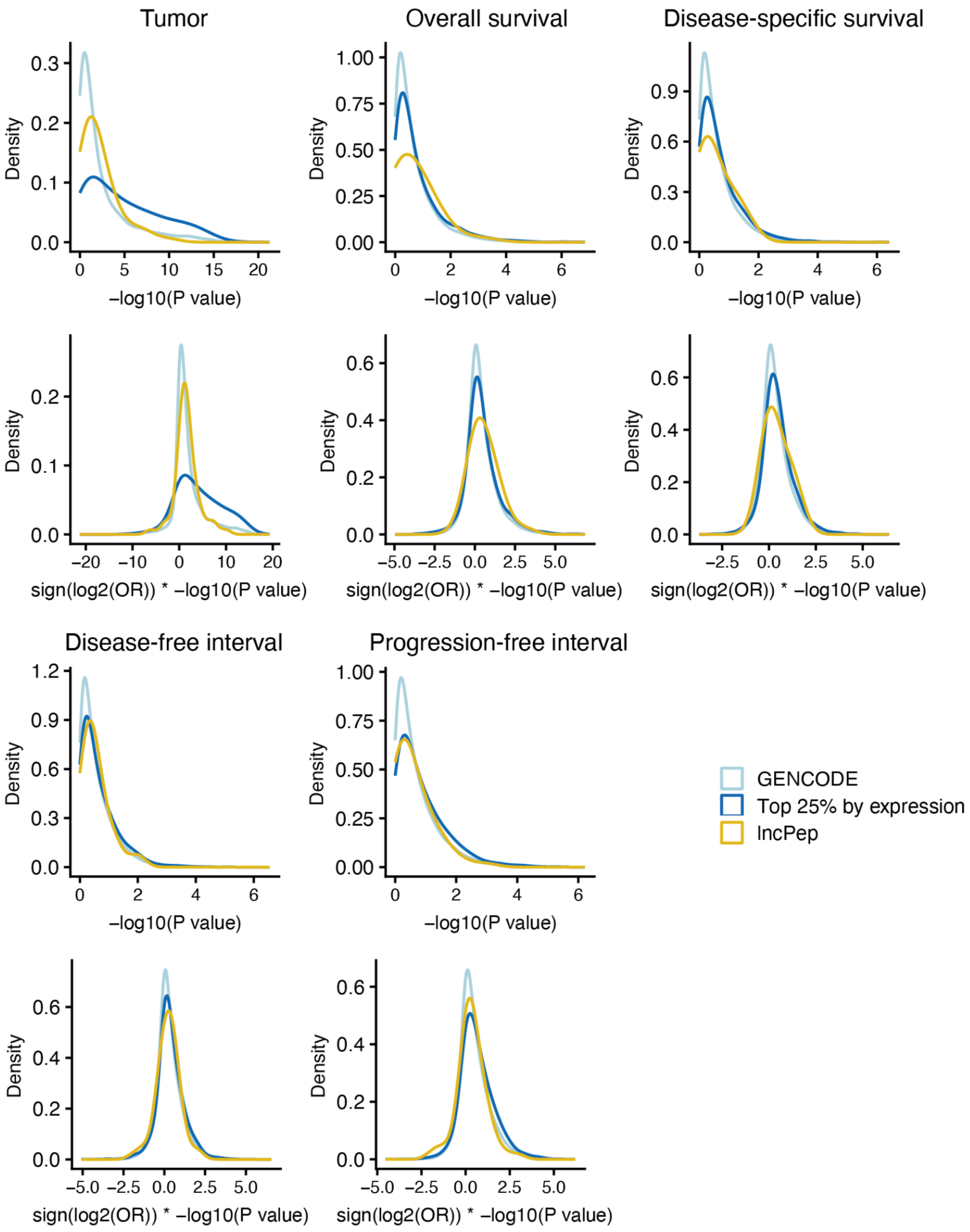

Figure S10: Supplementary figure for Figure 6

Density plots showing the distributions of odds ratios (ORs) for cancerous tissue and hazard ratios (HRs) for overall survival, disease-specific survival, disease-free interval, and progression-free interval for all lncRNAs annotated by GENCODE (v46), highly expressed lncRNAs (top 25% in TCGA-LIHC) and lncRNAs encoding lncPep discovered in this study.

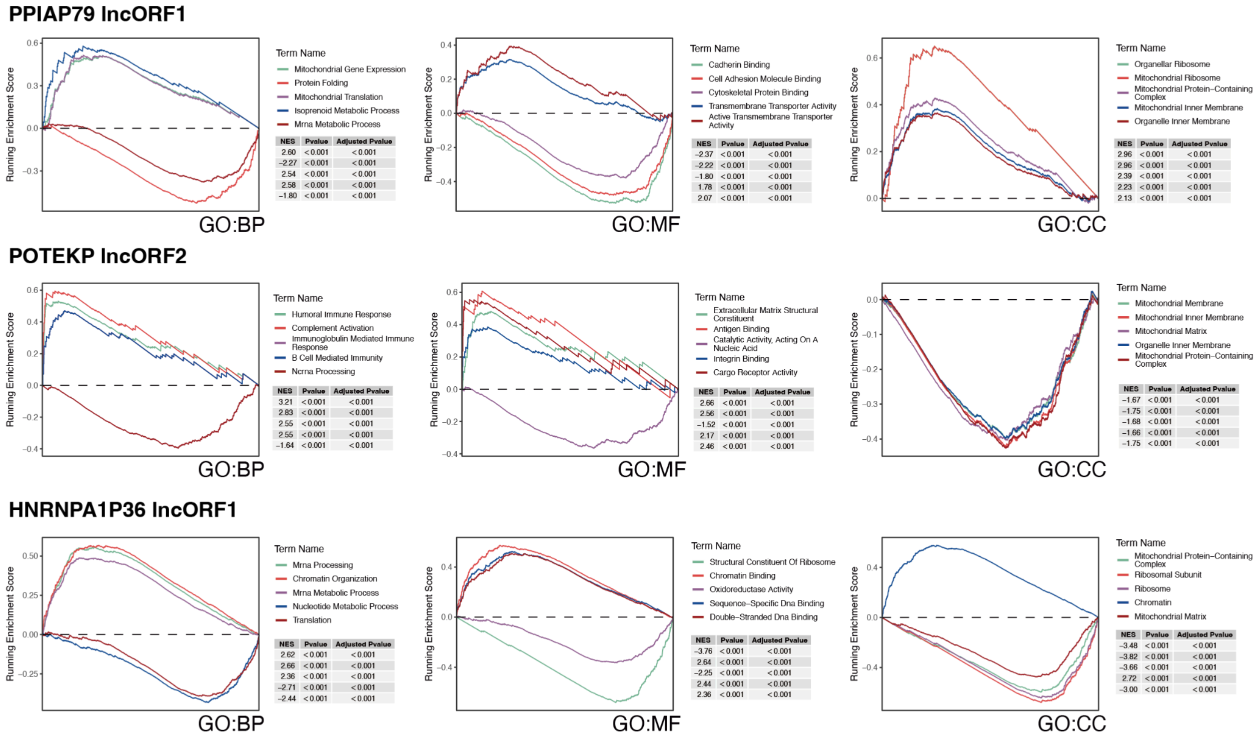

Figure S11: Supplementary figure for Figure 6

GSEA enrichment analysis of proteins with associated expression of *PPIAP79* lncORF1, *POTEKP* lncORF2, and *HNRNPA1P36* lncORF1. Pearson correlation coefficients ranked proteins with lncPep expression, and enrichment results are shown for Gene Ontology (GO) categories: Biological Process (BP), Molecular Function (MF), and Cellular Component (CC).
